# Supplementary material for: Identification of SNPs associated with magnesium and sodium uptake and the effect of their accumulation on micro and macro nutrient levels in Vitis vinifera
Source: PeerJ. 2021 Feb 8;9:e10773. doi: 10.7717/peerj.10773 (PMC7877238; doi:10.7717/peerj.10773)
Supplement: Supplemental Information 1 [file peerj-09-10773-s001.pdf]

Supplemental Figure 1. Histograms for untransformed nutrient concentrations across 249 *Vitis vinifera* F1 individuals.

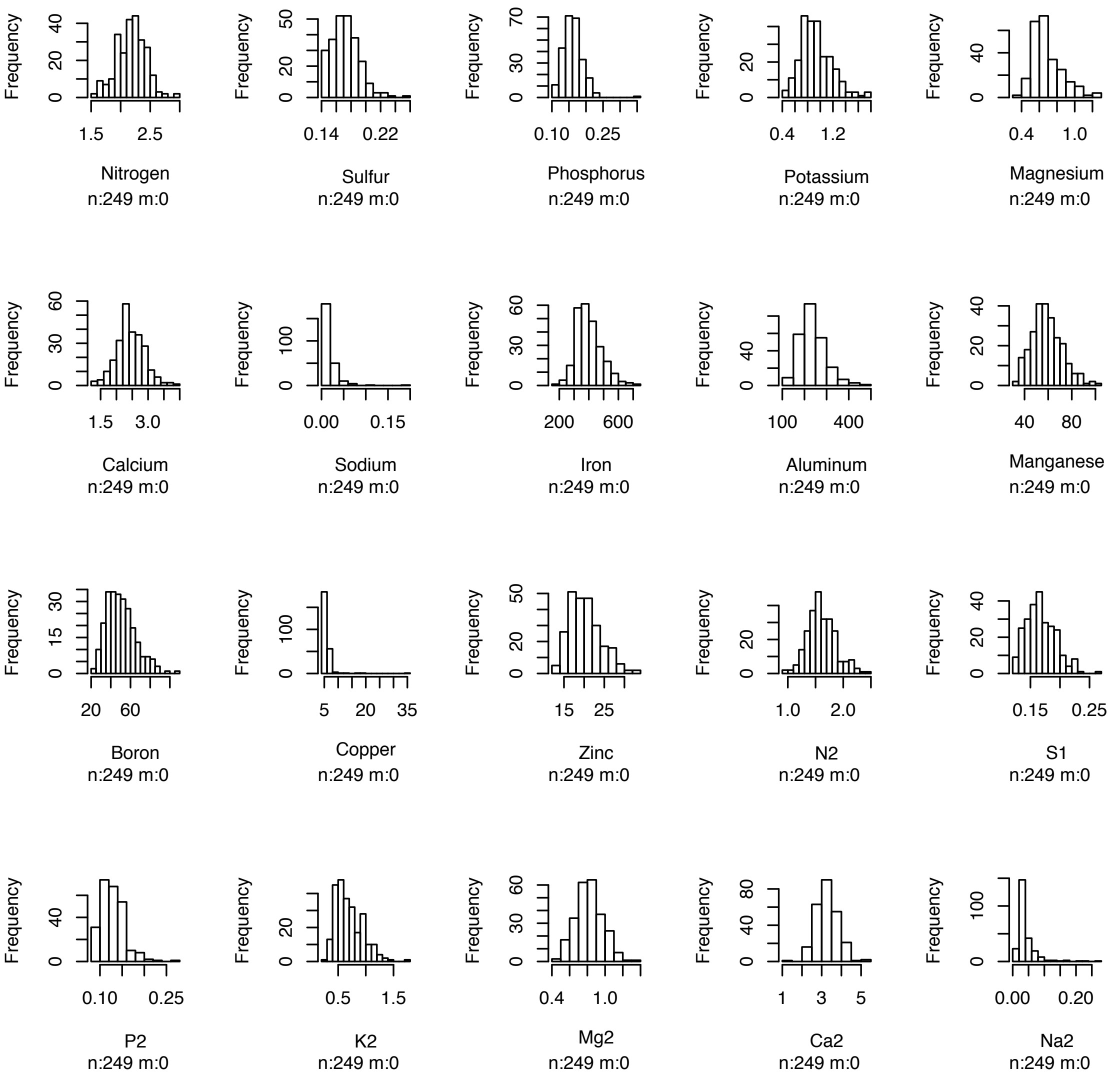

Supplemental Figure 2. Hierarchical Clustering of concentration B (1), Al (2), Fe (3), Na (4), Ca (5), and Mg (6) and presence of symptoms (red = symptomatic, gray = asymptomatic) (7) in A. 2015 and B. 2016. Each line represents the nutrient concentration of an individual from low (bright green) to high (red.).

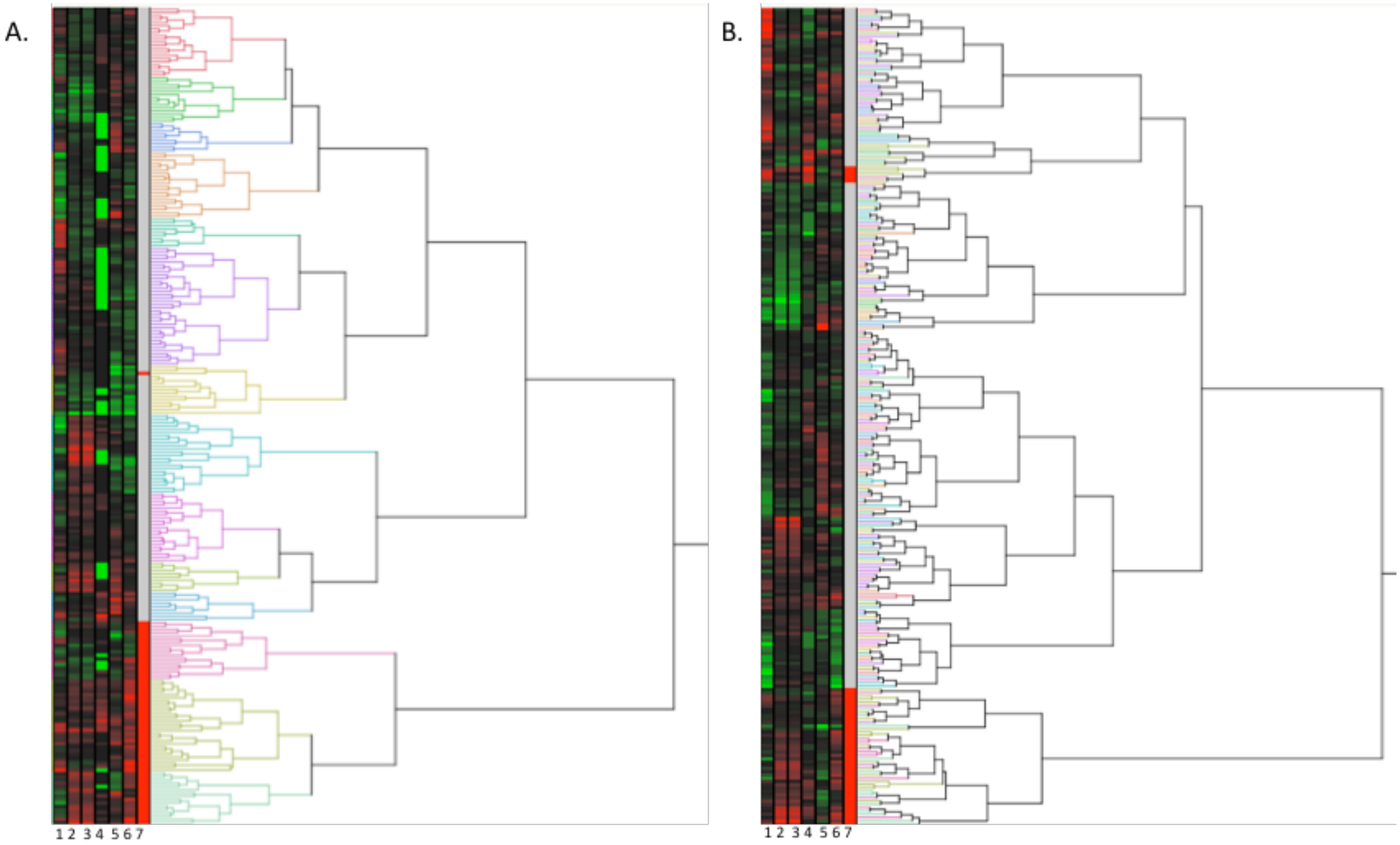

Supplemental Figure 3. Estimated linkage disequilibrium decay of the F_1 VxT population based on pairwise r^2 of SNPs aligned to the PN40024 genome.

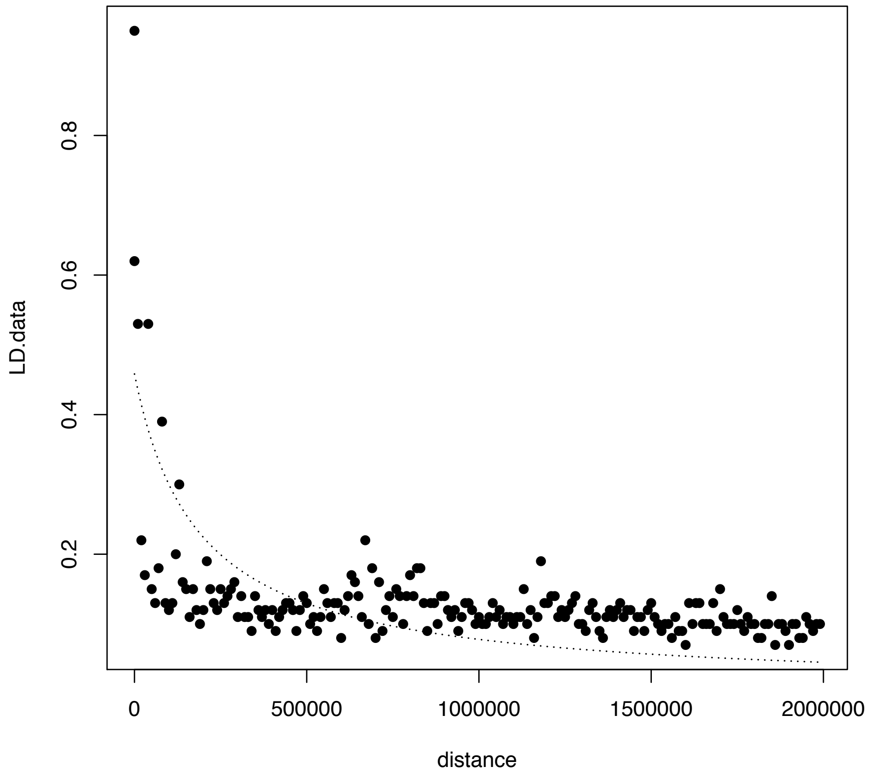

Supplemental Figure 4. Genome-wide associations across the Thompson Seedless genome for each of the phenotypic traits evaluated. Green lines denoting significance are visible on all nutrients and years where significant SNPs were detected. For those without a significance threshold, no significant SNPs were detected and the scale bar did not extend high enough to include it.

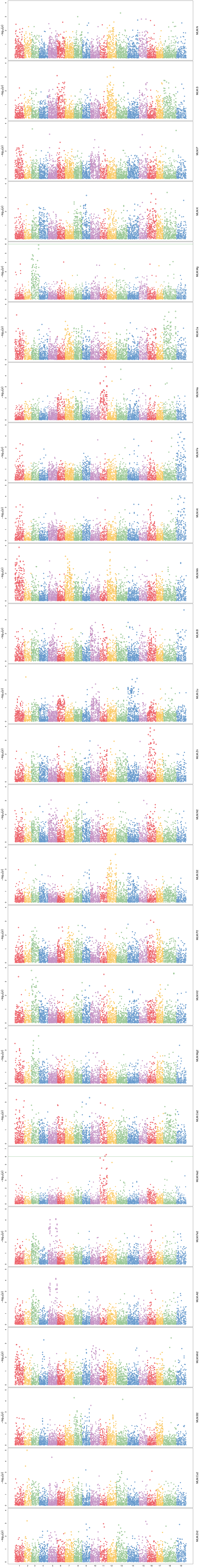

Supplemental Figure 5. BLAST2GO molecular function grouping for genes identified with SNPs aligned to the Thompson Seedless genome and associated with symptoms in 2015 and 2016.

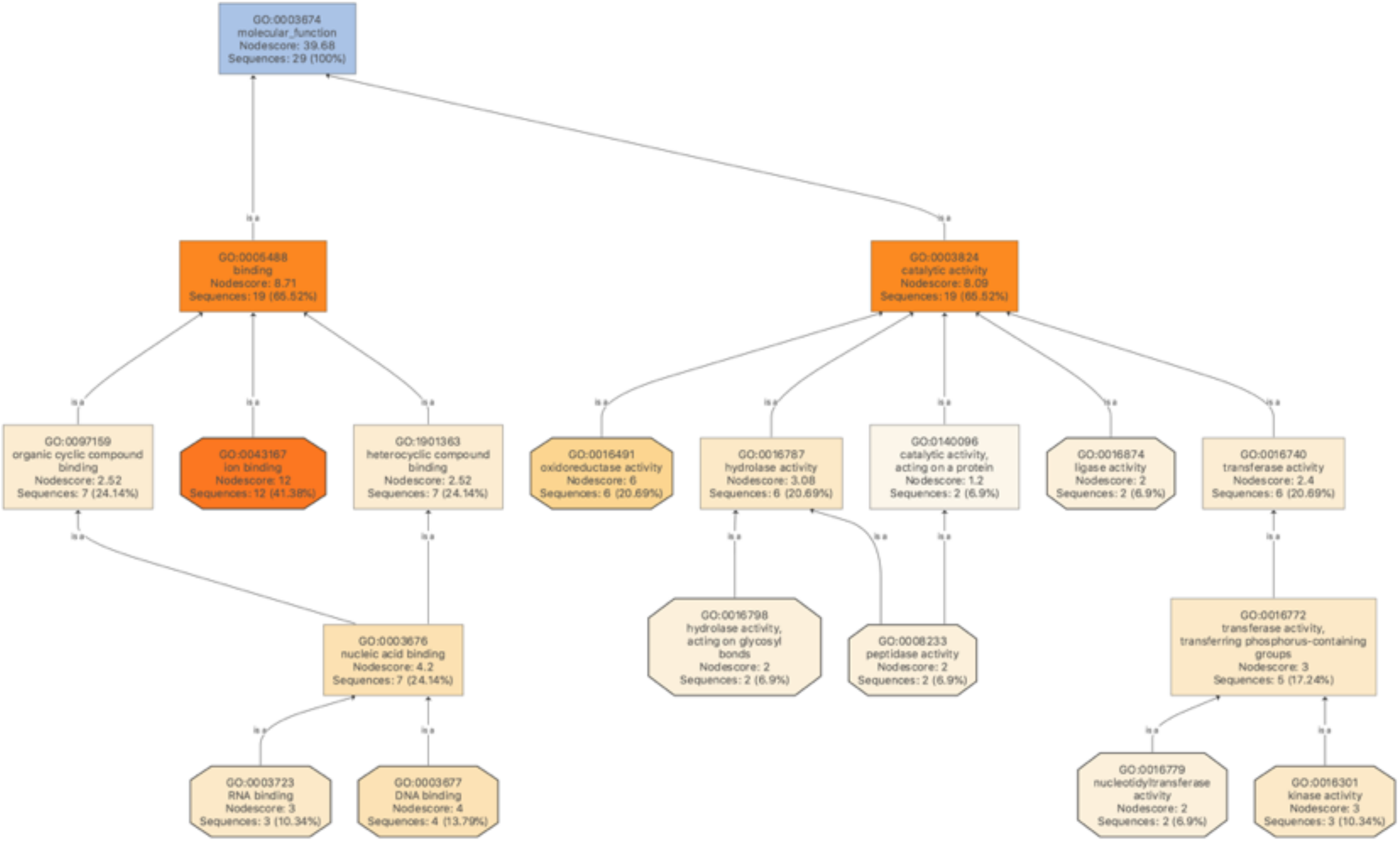

Supplemental Table 1: Mean Best Linear Unbiased Predictors (BLUPs) averaged across 2015 and 2016 for each nutrient concentration measured across 249 *Vitis vinifera* F1 individuals.

Individual	Nitrogen	Potassium	Aluminum	Magnesium	Boron	Calcium	Sulfur	Sodium	Iron	Phosphorus	Zinc	Plant Stunting	Marginal Chlorosis	Symptoms
Mg_1001	-0.11	0.14	-16.55	4.65	12.68	-0.03	0.00	-0.01	-23.86	-0.01	0.01	-0.18	-0.15	-0.19
Mg_1002	-0.04	0.22	2.15	-2.87	-5.35	-0.09	0.00	0.00	6.42	0.01	1.73	-0.18	0.27	0.24
Mg_1004	-0.02	0.03	3.68	-1.41	-6.26	-0.14	0.00	-0.01	2.97	0.00	-0.27	-0.18	-0.15	-0.19
Mg_1005	-0.01	-0.10	-17.12	-0.44	-12.27	-0.16	-0.01	-0.01	-31.84	0.00	0.30	-0.18	-0.15	-0.19
Mg_1006	-0.05	0.22	-13.12	5.86	3.96	0.03	0.01	-0.01	-15.70	0.00	-0.27	-0.18	-0.15	-0.19
Mg_1007	-0.05	0.38	9.21	3.92	-3.85	-0.04	0.00	-0.01	14.03	0.02	1.73	-0.18	-0.15	-0.19
Mg_1008	0.02	0.28	-7.96	9.25	22.60	-0.06	0.03	0.00	-11.53	0.01	0.30	-0.18	-0.15	-0.19
Mg_1009	-0.18	0.16	-14.07	6.58	0.96	0.23	0.00	0.00	-22.23	0.00	0.16	-0.18	-0.15	-0.19
Mg_1010	-0.02	0.11	-14.64	4.16	4.57	0.02	0.01	0.00	-20.05	0.00	-0.70	-0.18	-0.15	-0.19
Mg_1012	-0.01	0.17	-3.76	-3.11	-3.85	0.11	0.01	0.00	-6.82	0.02	-0.27	0.68	0.70	0.68
Mg_1013	0.09	-0.01	26.77	-4.08	0.66	-0.12	0.02	0.01	33.07	0.01	0.59	0.68	0.70	0.68
Mg_1014	-0.04	-0.04	15.89	4.89	10.58	0.35	0.01	-0.01	23.64	0.00	1.16	-0.18	-0.15	-0.19
Mg_1015	-0.01	0.05	-7.96	-0.69	5.47	-0.02	0.00	-0.01	-14.07	0.01	2.44	-0.18	-0.15	-0.19
Mg_1016	-0.02	0.14	5.40	1.01	24.10	-0.15	0.00	-0.01	-0.29	0.00	0.16	-0.18	-0.15	-0.19
Mg_1017	0.09	-0.05	20.85	7.80	22.90	0.13	0.00	-0.01	30.89	0.00	-0.13	-0.18	-0.15	-0.19
Mg_1018	-0.04	0.14	-17.31	4.16	-11.07	0.03	-0.01	-0.01	-29.12	0.00	0.30	-0.18	-0.15	-0.19
Mg_1019	0.08	0.09	1.77	9.25	17.79	-0.16	0.01	-0.01	-13.53	0.04	1.87	-0.18	-0.15	-0.19
Mg_1020	0.07	0.02	-24.38	-1.90	16.29	-0.12	0.01	0.01	-39.27	0.01	-0.13	-0.18	-0.15	-0.19
Mg_1021	-0.16	0.08	29.82	1.25	3.66	0.07	0.02	-0.01	39.23	0.00	-1.41	0.68	0.70	0.68
Mg_1022	-0.12	-0.08	19.71	-5.29	7.27	0.09	0.01	-0.01	18.38	0.00	0.44	0.68	0.70	0.68
Mg_1023	0.09	0.13	-2.43	-1.66	-0.54	-0.21	0.00	-0.01	-12.08	0.01	1.73	-0.18	-0.15	-0.19
Mg_1024	-0.02	0.15	40.13	1.98	-1.75	-0.29	0.00	-0.01	57.73	0.00	0.01	-0.18	-0.15	-0.19
Mg_1025	-0.03	0.05	118.37	1.74	-3.25	-0.18	0.02	-0.01	178.12	0.00	0.16	-0.18	-0.15	-0.19
Mg_1026	-0.03	0.01	26.58	4.65	-1.15	0.04	0.00	0.00	37.60	-0.01	-0.70	-0.18	-0.15	-0.19
Mg_1027	-0.04	-0.11	71.05	4.16	1.56	0.27	0.01	0.00	99.79	0.00	-0.98	-0.18	-0.15	-0.19
Mg_1028	0.14	-0.09	8.07	4.89	13.28	0.00	0.01	0.02	6.42	0.01	0.30	-0.18	-0.15	-0.19
Mg_1029	0.05	0.07	40.89	3.43	-3.25	-0.09	0.00	0.00	61.72	0.01	-1.27	0.68	0.70	0.68
Mg_1030	0.08	0.24	52.15	0.28	0.06	-0.35	0.00	0.01	82.39	0.01	-0.27	0.68	0.70	0.68
Mg_1032	0.27	0.24	39.75	2.46	4.57	-0.21	0.02	0.00	71.15	0.01	1.87	-0.18	-0.15	-0.19
Mg_1033	0.04	0.05	45.28	9.73	-0.24	-0.02	0.00	0.00	79.67	0.00	0.59	0.25	0.27	0.24
Mg_1034	0.05	0.12	19.52	-3.84	2.76	-0.23	0.00	-0.01	31.44	0.00	-1.13	-0.18	0.27	0.24
Mg_1035	0.06	0.18	15.13	1.25	2.76	-0.19	0.00	0.01	32.89	0.00	-0.27	0.25	0.27	0.24
Mg_1036	0.13	0.04	6.73	-5.05	4.87	-0.15	0.01	-0.01	15.66	0.00	-1.27	-0.18	-0.15	-0.19
Mg_1037	0.07	0.04	7.49	0.28	-5.65	0.05	0.01	-0.01	10.95	0.01	-0.27	-0.18	-0.15	-0.19

Mg_1038	0.01	-0.11	-12.73	-4.32	-13.17	-0.12	0.00	0.01	-18.24	0.00	-1.70	-0.18	-0.15	-0.19
Mg_1040	0.19	0.05	7.30	4.89	-3.25	-0.23	0.00	0.01	10.77	0.00	0.30	-0.18	-0.15	-0.19
Mg_1041	-0.04	-0.10	32.88	3.43	-3.85	0.19	-0.01	-0.01	48.48	-0.01	-1.56	-0.18	-0.15	-0.19
Mg_1042	0.03	-0.07	-2.43	3.19	-3.85	0.29	0.00	0.00	-5.19	0.00	2.01	-0.18	-0.15	-0.19
Mg_1043	0.10	-0.09	0.43	-0.69	8.47	0.02	0.00	0.00	-0.84	0.00	-0.56	-0.18	-0.15	-0.19
Mg_1044	0.00	-0.04	-15.60	16.52	-4.75	0.20	0.00	-0.01	-19.15	-0.01	-0.84	-0.18	-0.15	-0.19
Mg_1045	-0.02	-0.07	-9.87	3.92	-0.24	0.10	0.00	-0.01	-12.98	0.00	-1.70	-0.18	-0.15	-0.19
Mg_1046	-0.06	-0.17	0.82	3.19	1.86	0.23	0.00	-0.01	9.50	-0.01	-1.84	-0.18	-0.15	-0.19
Mg_1047	0.03	-0.10	65.70	1.74	9.68	-0.05	0.00	0.00	94.90	0.00	-0.27	-0.18	-0.15	-0.19
Mg_1048	0.08	0.04	13.41	-7.71	-9.26	-0.13	0.00	-0.01	16.57	0.00	-0.27	-0.18	-0.15	-0.19
Mg_1050	0.04	0.03	21.04	-6.99	-5.96	-0.10	0.00	-0.01	29.26	0.00	-1.84	0.68	0.70	0.68
Mg_1052	-0.05	-0.09	-27.24	-3.11	3.96	0.11	-0.01	-0.01	-32.93	0.00	-0.41	-0.18	-0.15	-0.19
Mg_1053	-0.05	0.02	-18.46	7.31	-2.95	0.05	-0.01	-0.01	-18.79	-0.01	-1.27	-0.18	-0.15	-0.19
Mg_1054	-0.05	0.10	-8.54	1.74	9.68	0.04	0.00	0.01	-13.89	0.01	-0.56	0.68	0.70	0.68
Mg_1055	-0.06	0.07	-1.67	-8.44	-1.15	0.01	-0.01	0.00	5.69	-0.01	-0.98	0.68	0.70	0.68
Mg_1056	-0.05	-0.17	17.04	-0.44	-4.45	0.21	0.00	-0.01	26.54	-0.01	0.44	-0.18	-0.15	-0.19
Mg_1057	0.10	-0.01	-10.83	2.46	-6.86	0.16	0.03	0.00	-13.16	0.00	0.30	-0.18	-0.15	-0.19
Mg_1058	-0.07	-0.09	-34.87	-4.81	-1.75	0.05	0.00	-0.01	-51.06	-0.01	-1.98	-0.18	-0.15	-0.19
Mg_1059	0.07	-0.15	-37.54	0.04	0.06	0.21	0.01	0.03	-51.60	0.00	0.30	-0.18	-0.15	-0.19
Mg_1060	-0.05	-0.19	-23.42	2.46	2.76	0.50	0.00	0.00	-28.58	-0.01	-1.84	-0.18	-0.15	-0.19
Mg_1061	-0.07	-0.15	-22.85	-3.35	0.36	0.09	-0.01	-0.01	-28.39	-0.01	-2.27	-0.18	-0.15	-0.19
Mg_1062	0.00	-0.17	-19.80	-3.59	-4.45	0.41	0.00	0.00	-22.77	0.00	-0.84	-0.18	-0.15	-0.19
Mg_1063	0.04	0.07	-11.21	1.98	1.86	0.18	0.01	-0.01	-9.90	0.00	0.16	-0.18	-0.15	-0.19
Mg_1064	-0.14	-0.03	0.05	-1.90	-11.97	0.03	-0.01	-0.01	0.62	-0.01	-0.41	-0.18	-0.15	-0.19
Mg_1065	0.01	-0.08	-19.60	-4.32	-8.36	0.07	0.00	0.00	-30.93	0.00	-0.98	-0.18	-0.15	-0.19
Mg_1067	0.06	-0.06	31.16	1.74	6.37	-0.12	0.01	0.07	41.77	0.01	2.01	0.68	0.70	0.68
Mg_1068	-0.03	-0.09	-10.64	-2.87	-3.25	0.01	-0.01	0.01	-23.50	0.00	-0.27	-0.18	-0.15	-0.19
Mg_1069	-0.01	-0.05	-3.96	-8.68	-2.65	-0.11	0.00	0.00	-4.28	0.01	0.16	-0.18	-0.15	-0.19
Mg_1070	0.02	-0.04	16.85	-6.74	-7.16	-0.24	-0.01	0.00	29.44	-0.01	-0.98	-0.18	-0.15	-0.19
Mg_1072	0.06	-0.02	-5.29	2.22	10.88	-0.23	-0.01	0.00	-5.55	0.00	-0.70	-0.18	-0.15	-0.19
Mg_1073	0.11	0.10	40.13	-2.38	-11.07	-0.43	0.00	0.00	59.72	0.01	-1.27	0.68	0.70	0.68
Mg_1074	0.02	0.01	-15.60	7.31	-1.75	0.06	0.00	0.00	-6.64	0.00	1.44	-0.18	-0.15	-0.19
Mg_1075	0.10	0.08	-17.31	1.01	-5.65	-0.21	0.00	-0.01	-19.15	-0.01	-0.84	-0.18	-0.15	-0.19
Mg_1076	-0.06	-0.11	-15.22	-0.69	-9.86	0.13	0.00	-0.01	-25.31	-0.01	-1.13	-0.18	-0.15	-0.19
Mg_1077	0.12	-0.12	-33.54	-6.74	-3.85	-0.06	-0.01	-0.01	-48.52	-0.01	-1.98	-0.18	-0.15	-0.19
Mg_1078	0.06	0.15	-32.77	-3.59	5.47	-0.05	0.00	0.05	-40.00	0.00	-0.70	0.25	0.27	0.24
Mg_1079	0.05	0.13	-36.02	-7.47	-6.86	-0.40	-0.01	0.00	-53.05	0.00	-1.27	-0.18	-0.15	-0.19
Mg_1080	0.16	0.06	14.56	-7.71	-2.35	-0.19	0.01	0.00	21.28	0.00	-1.41	-0.18	-0.15	-0.19
Mg_1081	-0.06	0.03	25.43	-3.11	-1.15	0.09	-0.01	0.02	27.81	0.00	2.58	0.68	0.70	0.68

Mg_1082	0.01	0.05	56.92	-0.93	-2.35	-0.17	0.00	0.02	78.94	0.01	0.30	0.68	0.70	0.68
Mg_1083	0.07	-0.07	-13.69	-6.26	0.36	-0.06	0.00	0.00	-17.15	-0.01	-0.98	-0.18	-0.15	-0.19
Mg_1084	0.01	-0.05	28.11	-0.93	-6.26	0.09	0.01	0.00	50.66	0.00	-0.84	-0.18	-0.15	-0.19
Mg_1085	-0.02	0.14	9.02	-0.20	-7.16	-0.14	0.01	0.00	17.84	0.00	0.16	-0.18	-0.15	-0.19
Mg_1086	0.04	0.03	24.48	0.28	-1.75	-0.06	0.01	0.00	38.69	0.00	0.16	-0.18	-0.15	-0.19
Mg_1087	0.12	-0.01	-24.57	-3.35	-4.15	-0.11	0.00	-0.01	-38.91	0.00	-1.27	-0.18	-0.15	-0.19
Mg_1088	0.00	-0.06	0.24	-0.69	3.66	0.13	0.00	0.00	-2.10	0.00	-0.56	-0.18	-0.15	-0.19
Mg_1089	-0.05	-0.02	-38.69	7.80	-8.06	-0.06	-0.01	-0.01	-51.60	0.00	0.44	0.25	-0.15	0.24
Mg_1090	-0.17	-0.06	37.08	0.77	2.76	0.03	-0.01	0.00	49.03	-0.01	0.30	0.68	0.70	0.68
Mg_1091	0.05	-0.01	0.24	-4.81	-1.75	0.00	0.00	0.00	0.80	0.02	0.30	-0.18	-0.15	-0.19
Mg_1092	-0.03	-0.17	-2.62	-1.66	-8.96	0.27	0.00	-0.01	-5.73	0.00	-0.27	0.25	-0.15	0.24
Mg_1093	-0.05	-0.11	17.23	-4.08	-7.16	-0.11	-0.01	-0.01	24.37	0.00	-1.13	0.25	0.27	0.24
Mg_1094	0.18	-0.08	-8.34	-8.68	-7.16	-0.39	-0.01	-0.01	-3.01	0.00	-1.41	-0.18	-0.15	-0.19
Mg_1096	-0.04	0.01	-24.76	-3.59	8.47	-0.05	0.00	0.00	-28.58	-0.01	-0.27	-0.18	-0.15	-0.19
Mg_1097	0.06	0.07	124.29	2.71	3.96	-0.31	0.00	0.00	189.90	0.02	0.01	0.68	0.70	0.68
Mg_1098	0.03	0.07	41.66	-1.17	-2.95	-0.26	0.00	0.00	61.54	0.01	-1.70	0.68	0.70	0.68
Mg_1099	-0.11	0.09	-15.02	-0.69	3.96	-0.11	-0.01	-0.01	-23.86	-0.01	-1.41	-0.18	-0.15	-0.19
Mg_1100	0.13	0.00	-11.21	-9.17	15.09	-0.31	0.00	0.06	-11.71	0.01	-0.13	-0.18	-0.15	-0.19
Mg_1101	0.04	-0.10	26.01	-3.35	18.09	0.12	0.00	0.01	34.70	0.00	0.44	0.25	-0.15	0.24
Mg_1102	0.12	-0.11	-13.50	-5.53	29.51	0.22	0.00	0.00	-18.24	0.00	0.87	-0.18	-0.15	-0.19
Mg_1103	0.17	0.02	28.87	-2.14	2.76	-0.29	0.00	0.01	44.49	0.01	-0.13	0.25	0.27	0.24
Mg_1104	0.01	-0.05	-1.47	0.53	-8.66	0.02	-0.01	0.01	1.88	0.00	0.87	-0.18	-0.15	-0.19
Mg_1105	0.12	0.03	-11.59	-5.78	-0.85	-0.33	0.00	-0.01	-1.20	-0.01	0.30	-0.18	-0.15	-0.19
Mg_1106	0.16	0.04	0.05	-14.01	-1.45	-0.53	-0.01	-0.01	18.38	0.01	-1.13	0.68	0.27	0.68
Mg_1107	0.10	-0.20	-4.91	-5.53	4.27	-0.20	-0.01	0.01	12.22	0.00	-1.13	-0.18	0.27	0.24
Mg_1108	0.04	-0.06	33.07	-5.29	-1.15	-0.18	0.00	0.00	48.12	0.00	0.59	0.68	0.27	0.68
Mg_1109	0.08	0.01	-13.50	-5.29	3.06	-0.07	0.01	0.01	-15.88	0.00	2.16	-0.18	-0.15	-0.19
Mg_1110	0.06	-0.06	-17.51	5.61	-5.05	0.07	0.00	0.01	-20.78	-0.01	0.16	-0.18	-0.15	-0.19
Mg_1111	-0.05	0.14	-3.19	-4.32	-5.05	-0.23	-0.01	0.00	3.88	0.00	0.16	-0.18	-0.15	-0.19
Mg_1112	-0.09	-0.15	-41.93	-3.11	-6.26	0.17	-0.02	-0.01	-61.76	-0.01	1.30	-0.18	-0.15	-0.19
Mg_1113	-0.04	-0.12	-33.15	-2.62	6.07	-0.05	-0.02	-0.01	-46.53	-0.01	-0.41	-0.18	-0.15	-0.19
Mg_1114	0.03	-0.15	-21.51	1.98	11.78	0.01	-0.01	-0.01	-31.11	0.00	0.59	-0.18	-0.15	-0.19
Mg_1115	0.04	-0.01	-24.18	-5.78	11.78	0.02	0.00	-0.01	-36.55	0.00	-0.70	-0.18	-0.15	-0.19
Mg_1116	0.08	0.06	-11.21	-10.14	-0.54	-0.16	0.00	-0.01	-12.62	0.00	0.16	-0.18	-0.15	-0.19
Mg_1117	0.05	0.05	-17.31	-7.23	-5.35	-0.09	0.00	-0.01	-18.42	0.00	-0.41	-0.18	-0.15	-0.19
Mg_1118	0.05	-0.12	-23.42	0.04	-1.45	0.21	0.00	-0.01	-31.66	-0.01	-0.84	-0.18	-0.15	-0.19
Mg_1119	0.02	0.02	-9.11	-3.59	8.17	0.13	0.00	0.01	-1.38	0.00	0.01	0.25	0.27	0.24
Mg_1120	0.08	-0.03	-17.31	-4.56	5.47	-0.13	0.00	-0.01	-17.15	0.00	-0.98	-0.18	-0.15	-0.19
Mg_1121	0.11	0.01	-21.89	1.74	16.59	-0.10	0.01	-0.01	-30.75	0.01	-0.98	-0.18	-0.15	-0.19

Mg_1122	0.09	-0.02	-23.80	-6.50	14.79	-0.16	0.00	0.02	-43.26	0.01	-0.27	-0.18	-0.15	-0.19
Mg_1123	0.02	-0.07	-6.05	-1.17	-3.25	0.26	0.00	0.03	-8.09	0.01	0.01	0.25	-0.15	0.24
Mg_1124	-0.04	0.04	-22.28	0.77	-3.85	0.04	0.00	0.00	-19.51	0.00	-0.13	0.25	-0.15	0.24
Mg_1125	-0.03	-0.06	-17.31	-1.66	-11.37	-0.05	0.00	0.00	-21.69	-0.01	1.01	-0.18	-0.15	-0.19
Mg_1126	0.01	-0.12	-6.82	-2.62	-2.65	0.04	-0.01	0.00	-11.90	0.01	0.44	-0.18	-0.15	-0.19
Mg_1127	-0.13	0.12	14.37	-6.99	-3.85	0.04	0.00	-0.01	19.47	0.00	-1.13	0.68	0.70	0.68
Mg_1128	0.18	0.03	-29.15	-12.80	0.36	-0.15	0.01	-0.01	-31.48	0.01	-1.41	-0.18	-0.15	-0.19
Mg_1129	-0.11	-0.09	69.14	14.82	15.09	0.02	-0.01	0.00	99.61	0.00	-0.84	0.68	0.70	0.68
Mg_1132	-0.09	0.33	-1.09	1.25	11.78	-0.30	-0.01	0.00	2.79	0.00	-0.41	-0.18	-0.15	-0.19
Mg_1133	-0.03	0.20	35.93	2.95	-8.36	-0.04	0.00	0.00	40.69	0.00	0.87	-0.18	-0.15	-0.19
Mg_1134	-0.16	0.08	23.14	2.22	-2.05	0.14	-0.01	0.01	41.05	-0.01	-0.41	0.25	0.27	0.24
Mg_1135	-0.01	-0.16	47.95	17.73	-10.16	0.15	0.00	0.00	64.62	-0.01	-0.13	-0.18	-0.15	-0.19
Mg_1136	0.01	-0.15	11.88	-4.08	-7.76	-0.09	0.00	0.00	14.21	-0.01	0.30	-0.18	-0.15	-0.19
Mg_1137	-0.01	-0.18	-11.97	4.89	4.87	-0.06	-0.01	-0.01	-24.77	0.00	-0.56	-0.18	-0.15	-0.19
Mg_1139	0.08	0.13	15.70	9.98	3.36	0.11	0.02	0.00	27.27	0.00	2.44	-0.18	-0.15	-0.19
Mg_1140	0.10	0.19	-11.59	1.74	10.28	-0.29	0.01	0.00	-3.74	0.00	0.30	-0.18	-0.15	-0.19
Mg_1141	0.07	0.12	-7.01	-2.38	4.57	-0.31	0.00	0.00	-7.73	0.01	-0.98	0.68	-0.15	0.68
Mg_1142	0.21	-0.04	-7.20	-3.84	-3.25	0.07	0.01	-0.01	-16.25	0.02	0.16	-0.18	-0.15	-0.19
Mg_1143	-0.07	-0.08	-1.28	-6.74	-3.85	-0.10	-0.01	0.00	-9.72	0.00	-0.98	0.68	0.70	0.68
Mg_1144	0.03	0.04	-23.61	-6.02	-4.15	-0.06	-0.01	-0.01	-41.99	0.02	-0.56	0.68	0.70	0.68
Mg_1145	0.00	0.35	58.26	8.28	-4.75	0.06	0.01	0.01	84.02	0.01	1.73	0.68	0.70	0.68
Mg_1146	0.09	0.02	24.67	-1.17	5.17	0.34	0.01	0.00	33.61	0.00	1.44	-0.18	-0.15	-0.19
Mg_1147	0.16	-0.04	-15.60	9.73	-4.45	0.12	0.01	-0.01	-19.33	0.00	0.59	-0.18	-0.15	-0.19
Mg_1148	-0.09	-0.17	14.75	13.37	0.36	0.36	-0.01	0.03	18.57	-0.01	0.01	0.25	-0.15	0.24
Mg_1149	-0.04	0.04	5.59	7.31	-2.35	-0.11	-0.01	0.00	11.68	0.00	0.73	0.68	0.70	0.68
Mg_1150	-0.16	0.03	5.97	1.98	13.58	-0.05	-0.01	0.02	-2.65	0.00	0.16	0.68	0.70	0.68
Mg_1151	0.02	-0.04	-13.69	2.22	2.16	0.08	0.00	0.00	-17.52	0.00	1.16	-0.18	-0.15	-0.19
Mg_1152	-0.12	-0.13	-16.74	-3.35	-7.46	0.06	0.00	0.01	-23.68	0.00	0.16	-0.18	-0.15	-0.19
Mg_1153	-0.03	-0.01	4.25	7.80	0.06	0.28	0.00	0.01	14.39	0.00	1.30	-0.18	-0.15	-0.19
Mg_1154	-0.08	-0.10	-1.67	10.22	-2.35	0.31	0.00	0.01	-0.47	0.00	-1.70	-0.18	-0.15	-0.19
Mg_1155	-0.06	0.02	8.07	-7.96	3.06	-0.19	0.00	0.00	9.32	-0.01	-1.27	0.25	0.27	0.24
Mg_1156	0.01	-0.10	-12.93	-3.84	-10.76	0.25	0.00	-0.01	-18.24	0.00	0.73	-0.18	-0.15	-0.19
Mg_1157	0.09	0.00	-14.64	-0.93	-7.16	0.21	0.01	-0.01	-4.82	0.01	0.73	-0.18	-0.15	-0.19
Mg_1158	-0.02	-0.10	-21.51	-0.93	-2.05	0.07	0.00	-0.01	-29.12	-0.01	-0.84	-0.18	-0.15	-0.19
Mg_1160	-0.05	0.26	16.08	3.43	4.87	0.04	0.00	-0.01	6.05	0.02	1.87	0.68	0.70	0.68
Mg_1161	-0.02	0.00	9.21	-3.11	3.36	0.08	0.00	0.00	10.77	0.00	-1.13	-0.18	-0.15	-0.19
Mg_1162	0.15	0.03	-21.32	-4.08	1.86	-0.36	0.00	-0.01	-28.94	0.01	-1.84	-0.18	-0.15	-0.19
Mg_1163	-0.02	0.00	-0.14	0.77	4.57	-0.13	0.00	0.03	21.83	0.00	6.01	0.68	0.27	0.68
Mg_1164	-0.03	0.12	-14.64	3.68	-5.65	0.10	0.01	0.00	-16.07	0.00	1.44	-0.18	-0.15	-0.19

Mg_1165	0.01	0.18	6.73	0.53	-4.75	0.02	0.01	0.00	12.04	0.00	0.59	0.68	0.27	0.68
Mg_1166	-0.06	-0.12	-3.96	-0.69	-0.24	0.19	0.00	0.00	-8.27	-0.01	-0.98	-0.18	-0.15	-0.19
Mg_1167	-0.02	-0.12	-23.23	2.95	-8.66	0.27	0.00	0.00	-35.47	0.00	-0.84	-0.18	-0.15	-0.19
Mg_1169	0.04	0.03	-21.13	21.85	-8.06	0.29	0.01	-0.01	-16.43	0.00	-0.56	-0.18	-0.15	-0.19
Mg_1170	-0.08	0.32	-16.36	7.07	-0.85	0.05	0.00	-0.01	-21.50	-0.01	-0.41	-0.18	-0.15	-0.19
Mg_1171	-0.11	0.04	-13.88	-1.66	1.86	0.22	0.00	-0.01	-28.58	0.00	-0.56	-0.18	-0.15	-0.19
Mg_1172	0.05	-0.06	-7.20	-4.81	17.79	0.11	0.01	0.00	-21.69	0.02	-0.27	-0.18	-0.15	-0.19
Mg_1173	-0.04	0.00	-34.68	-8.93	-7.76	-0.21	-0.01	-0.01	-61.21	-0.01	0.01	-0.18	-0.15	-0.19
Mg_1175	-0.06	-0.03	-10.44	-3.84	-7.76	-0.03	0.00	-0.01	-20.78	0.00	-1.13	-0.18	-0.15	-0.19
Mg_1176	-0.14	-0.09	-11.97	-1.41	2.46	0.17	-0.02	-0.01	-20.96	-0.01	-0.13	-0.18	-0.15	-0.19
Mg_1177	-0.07	-0.16	-29.15	20.16	-1.15	0.56	0.00	-0.01	-41.09	-0.01	0.44	-0.18	-0.15	-0.19
Mg_1178	-0.13	0.04	2.34	9.01	-10.76	0.39	0.00	-0.01	0.62	-0.01	0.44	-0.18	-0.15	-0.19
Mg_1179	-0.07	0.10	-32.96	-7.96	-8.36	-0.24	-0.01	-0.01	-46.89	0.01	1.01	0.25	-0.15	0.24
Mg_1180	-0.13	-0.11	-20.75	-2.38	-6.26	0.06	-0.01	-0.01	-39.09	0.00	0.30	0.25	-0.15	0.24
Mg_1181	0.01	-0.15	-5.48	8.77	3.36	0.28	0.00	0.00	-10.26	0.00	-0.70	-0.18	-0.15	-0.19
Mg_1182	-0.04	-0.09	-13.12	8.04	6.07	0.23	0.02	0.00	-15.88	0.00	-0.13	-0.18	-0.15	-0.19
Mg_1183	-0.06	-0.04	-1.09	1.74	4.57	0.36	0.00	0.00	-7.18	0.00	-0.56	-0.18	-0.15	-0.19
Mg_1184	0.02	-0.01	-38.31	-2.62	-4.15	0.16	0.00	-0.01	-59.58	0.00	-1.84	-0.18	-0.15	-0.19
Mg_1185	0.04	-0.12	-28.38	-5.29	-8.06	0.20	0.00	-0.01	-42.54	0.00	-0.70	-0.18	-0.15	-0.19
Mg_1187	-0.17	-0.14	-12.16	-0.93	-1.75	0.30	-0.01	0.00	-30.21	0.01	-0.41	-0.18	-0.15	-0.19
Mg_1188	-0.03	-0.02	-13.50	-7.23	11.18	-0.04	0.00	-0.01	-29.85	0.00	-0.13	-0.18	-0.15	-0.19
Mg_1189	-0.17	0.12	9.21	-1.66	2.16	0.01	0.00	0.00	4.24	0.01	-0.41	0.68	0.70	0.68
Mg_1190	-0.05	-0.03	1.96	-0.44	19.29	0.00	0.01	-0.01	-17.15	0.02	0.16	-0.18	-0.15	-0.19
Mg_1191	-0.05	0.14	-14.26	-1.90	5.17	0.08	0.00	-0.01	-26.94	0.01	1.73	-0.18	-0.15	-0.19
Mg_1192	0.00	0.01	-28.00	-5.78	17.79	-0.15	0.01	-0.01	-43.08	0.00	-0.84	-0.18	-0.15	-0.19
Mg_1193	0.02	-0.15	-34.11	-6.74	-4.75	-0.08	-0.01	-0.01	-57.22	-0.01	-0.84	-0.18	-0.15	-0.19
Mg_1195	-0.16	-0.12	82.50	5.13	-3.55	-0.06	0.00	0.01	116.11	-0.01	0.01	0.68	0.27	0.68
Mg_1196	0.01	-0.16	-7.77	-8.44	9.98	0.02	-0.01	-0.01	-19.69	0.00	-0.70	-0.18	-0.15	-0.19
Mg_1198	0.01	-0.07	-23.61	-1.41	-3.85	0.01	0.00	0.00	-36.37	0.00	1.01	-0.18	-0.15	-0.19
Mg_1199	0.05	0.02	-23.04	-7.47	-8.36	-0.13	0.01	0.00	-37.64	0.00	0.16	-0.18	-0.15	-0.19
Mg_1200	-0.14	-0.21	-26.67	9.49	-5.65	0.41	-0.02	-0.01	-44.53	-0.01	-0.84	-0.18	-0.15	-0.19
Mg_1201	0.22	0.14	-5.48	2.46	6.37	0.10	0.02	-0.01	-11.53	0.02	0.87	-0.18	-0.15	-0.19
Mg_1202	0.00	-0.07	-9.87	1.49	0.96	0.13	0.01	-0.01	-20.60	0.00	-1.27	-0.18	-0.15	-0.19
Mg_1203	0.08	-0.02	-4.91	3.68	-11.37	0.13	0.01	-0.01	-12.98	0.00	-0.13	-0.18	-0.15	-0.19
Mg_1204	-0.06	0.00	-10.44	-9.17	7.27	0.02	0.00	0.00	-32.02	0.01	0.01	0.25	0.27	0.68
Mg_1205	0.09	-0.09	13.03	-2.38	3.06	-0.04	0.00	0.01	11.13	0.00	-0.70	-0.18	-0.15	-0.19
Mg_1206	-0.03	0.05	-11.02	14.82	-7.46	0.19	0.00	0.01	-23.50	0.00	1.87	-0.18	-0.15	-0.19
Mg_1208	0.02	0.03	-12.16	-5.53	-2.65	-0.16	-0.01	0.00	-20.60	0.00	0.16	-0.18	-0.15	-0.19
Mg_1209	-0.01	0.11	7.69	-6.02	-8.06	-0.15	0.00	0.01	7.14	0.00	-0.70	-0.18	-0.15	-0.19

Mg_1210	0.01	-0.02	-1.86	-0.44	-3.55	0.02	0.00	0.02	-1.74	0.01	0.01	-0.18	-0.15	-0.19
Mg_1211	-0.04	0.33	17.04	-7.47	-6.56	-0.53	-0.01	0.01	23.64	0.00	-0.41	0.68	0.27	0.68
Mg_1212	-0.08	0.05	36.69	2.71	19.90	-0.26	-0.01	0.06	61.17	-0.01	1.87	0.68	0.27	0.68
Mg_1213	0.02	-0.05	-9.11	-1.90	-8.06	-0.02	-0.01	0.00	-11.90	0.00	1.73	-0.18	-0.15	-0.19
Mg_1214	-0.12	-0.10	14.17	-0.69	-3.25	-0.17	-0.01	-0.01	22.92	-0.01	-1.41	0.68	0.70	0.68
Mg_1215	-0.25	-0.21	-21.70	5.13	7.57	0.27	-0.01	0.01	-25.31	-0.02	0.01	0.25	-0.15	0.24
Mg_1216	-0.04	0.03	-16.36	-4.08	1.26	-0.31	-0.02	-0.01	-28.58	-0.01	-0.98	0.68	0.70	0.68
Mg_1217	-0.23	-0.23	-14.26	-0.69	6.67	0.10	-0.02	0.00	-21.14	-0.02	-1.98	-0.18	-0.15	-0.19
Mg_1219	-0.06	-0.15	-31.06	6.83	2.76	0.03	-0.01	0.00	-39.27	-0.01	0.16	-0.18	-0.15	-0.19
Mg_1223	-0.02	-0.06	-7.96	-4.32	-2.65	-0.09	0.01	-0.01	-13.16	0.00	-0.13	-0.18	-0.15	-0.19
Mg_1229	0.06	-0.02	7.49	-4.32	2.76	0.12	0.02	0.00	7.69	0.01	0.59	-0.18	-0.15	-0.19
Mg_1239	-0.03	0.08	23.33	10.22	0.66	0.15	0.02	-0.01	35.43	0.00	-0.41	-0.18	-0.15	-0.19
Mg_1241	-0.09	0.16	34.59	3.19	-2.95	-0.03	0.00	-0.01	44.13	0.00	-0.70	0.68	0.70	0.68
Mg_1242	0.12	0.18	26.01	4.89	-14.07	-0.20	0.00	0.00	41.23	-0.01	-0.13	-0.18	-0.15	-0.19
Mg_1243	0.08	0.09	5.01	22.58	-5.65	0.00	0.02	-0.01	6.24	-0.01	0.73	-0.18	-0.15	-0.19
Mg_1244	0.04	-0.06	16.66	19.19	-7.76	0.14	0.01	-0.01	18.93	0.00	0.44	-0.18	-0.15	-0.19
Mg_1245	0.09	0.16	6.73	6.10	-4.45	-0.11	0.01	-0.01	15.85	-0.01	0.16	-0.18	-0.15	-0.19
Mg_1246	0.07	0.03	-5.29	-2.87	0.06	-0.33	0.00	-0.01	-7.36	-0.01	-0.98	-0.18	-0.15	-0.19
Mg_1247	-0.14	0.17	43.18	8.77	20.20	-0.18	0.00	-0.01	55.01	-0.01	0.30	0.68	0.70	0.68
Mg_1248	-0.11	-0.13	20.47	1.01	-0.54	0.02	0.00	0.01	30.35	-0.01	-0.56	-0.18	-0.15	-0.19
Mg_1249	0.04	-0.11	0.24	1.74	6.97	-0.03	0.00	-0.01	5.15	-0.01	-0.13	-0.18	-0.15	-0.19
Mg_1250	0.00	0.21	7.30	-4.81	2.76	-0.13	0.00	0.01	8.05	-0.01	-0.27	-0.18	-0.15	-0.19
Mg_1251	0.00	0.18	-15.98	-3.84	-12.27	-0.03	0.00	0.00	-22.41	0.01	0.73	-0.18	-0.15	-0.19
Mg_1252	-0.03	0.06	27.72	7.55	3.06	0.17	0.00	-0.01	41.41	0.00	1.73	-0.18	-0.15	-0.19
Mg_1253	0.03	0.11	-21.51	8.04	0.06	-0.04	0.00	-0.01	-17.33	-0.01	0.73	-0.18	-0.15	-0.19
Mg_1254	-0.12	0.12	29.82	-8.68	15.09	-0.07	0.00	0.04	37.06	0.00	-0.84	0.68	0.27	0.68
Mg_1261	0.15	-0.02	-11.02	1.49	-2.35	-0.20	0.01	-0.01	-19.51	0.00	0.87	-0.18	-0.15	-0.19
Mg_1262	0.00	0.08	49.67	1.98	-5.05	-0.12	0.00	0.00	68.06	0.02	-0.41	0.68	0.70	0.68
Mg_1264	-0.03	-0.04	42.80	0.04	-3.85	-0.20	0.00	0.01	59.36	0.00	-0.13	0.68	0.70	0.68
Mg_1265	-0.04	0.08	84.79	5.86	-0.54	-0.18	0.02	0.01	126.63	0.00	0.16	0.25	0.70	0.68
Mg_1267	0.06	0.07	28.11	7.80	3.66	0.02	0.02	0.00	45.58	0.01	0.44	-0.18	-0.15	-0.19
Mg_1268	0.05	-0.02	32.69	-2.87	-2.05	-0.13	0.00	-0.01	44.49	0.00	-0.13	-0.18	-0.15	-0.19
Mg_1270	-0.06	0.02	-9.30	-6.02	-11.07	-0.12	0.00	-0.01	-9.72	0.00	0.01	-0.18	-0.15	-0.19
Mg_1274	-0.04	0.01	37.46	-6.02	-8.36	-0.23	0.00	0.00	55.01	-0.01	-0.27	0.25	0.70	0.68
Mg_1275	0.02	-0.17	-17.12	6.34	8.47	0.31	-0.01	-0.01	-22.59	-0.01	-1.13	-0.18	-0.15	-0.19
Mg_1276	0.03	-0.06	-8.15	0.77	1.26	0.18	0.00	0.00	-16.43	0.00	0.30	-0.18	-0.15	-0.19
Mg_1277	0.14	0.09	-8.73	-2.62	-2.95	-0.22	0.00	0.00	-15.88	0.01	0.73	-0.18	-0.15	-0.19
Mg_1278	-0.03	-0.07	-0.71	-7.23	-3.55	-0.02	-0.01	-0.01	-12.62	0.00	1.30	-0.18	-0.15	-0.19
Mg_1279	-0.08	-0.11	-27.81	24.03	-8.36	0.42	-0.01	0.01	-41.63	-0.02	2.16	-0.18	-0.15	-0.19

Mg_1280	-0.03	-0.02	-15.60	5.86	-8.96	0.29	-0.01	-0.01	-28.94	-0.01	0.73	-0.18	-0.15	-0.19
Mg_1281	0.15	-0.06	-41.55	-3.59	3.36	-0.23	0.00	0.00	-62.48	0.01	1.01	-0.18	-0.15	-0.19
Mg_1282	-0.06	0.04	33.26	2.22	2.76	-0.32	-0.01	0.00	41.23	0.01	0.16	0.68	0.70	0.68
Mg_1283	0.06	0.05	-16.93	-2.14	5.77	-0.08	0.00	-0.01	-27.31	-0.01	0.44	-0.18	-0.15	-0.19
Mg_1284	-0.01	0.00	30.59	1.01	-4.15	0.33	0.00	-0.01	40.69	0.00	0.44	-0.18	-0.15	-0.19
Mg_1285	0.01	-0.09	3.68	0.53	3.66	0.10	0.00	-0.01	7.14	0.00	-0.56	-0.18	-0.15	-0.19
Mg_1286	-0.02	-0.09	-16.93	-3.84	-0.85	-0.03	-0.01	-0.01	-32.38	0.00	1.30	-0.18	-0.15	-0.19
Mg_1287	0.05	-0.10	-50.52	-5.29	-10.76	-0.05	-0.01	-0.01	-75.72	-0.01	0.87	-0.18	-0.15	-0.19
Mg_1289	-0.15	-0.09	20.47	-9.90	-0.54	0.04	-0.01	0.01	18.75	0.00	3.15	0.68	0.70	0.68
Mg_1290	0.01	-0.03	-22.28	-9.41	-6.86	-0.19	-0.01	0.00	-32.75	0.01	1.44	-0.18	-0.15	-0.19
Mg_1291	0.02	0.12	1.39	-6.74	-9.26	-0.12	0.00	-0.01	-5.01	0.02	-0.41	-0.18	-0.15	-0.19
Mg_1292	-0.15	-0.01	-4.91	1.98	-1.45	0.34	0.01	-0.01	-6.46	-0.01	1.30	-0.18	-0.15	-0.19
Mg_1293	0.03	-0.03	-21.32	3.68	-2.65	0.27	0.00	0.00	-24.59	0.00	1.44	-0.18	-0.15	-0.19
Mg_1294	-0.02	0.03	-9.11	-6.02	-7.76	0.01	0.00	0.00	-12.44	0.00	1.73	-0.18	-0.15	-0.19
Mg_1295	0.06	-0.16	-40.02	-9.65	-7.46	0.48	0.00	0.00	-50.15	0.00	2.30	-0.18	-0.15	-0.19
Mg_1296	-0.05	-0.20	-24.38	-11.11	-2.05	0.00	-0.01	0.00	-38.91	-0.01	-0.56	-0.18	-0.15	-0.19
Mg_1297	-0.03	-0.08	-37.73	-1.90	-11.67	0.25	0.00	-0.01	-55.05	0.00	1.30	-0.18	-0.15	-0.19
Mg_1298	-0.11	-0.11	-32.58	-4.08	-9.86	0.19	0.00	-0.01	-46.16	-0.01	-0.27	-0.18	-0.15	-0.19
Mg_1299	-0.01	-0.06	-53.77	-13.05	-7.46	-0.12	-0.01	-0.01	-83.51	-0.01	-0.70	-0.18	-0.15	-0.19
Mg_1300	-0.07	-0.15	-31.63	6.83	8.47	0.18	-0.01	0.00	-42.17	-0.01	2.73	-0.18	-0.15	-0.19

1

Supplemental Table 2: Pearson's Correlation Coefficients for nutrients measures in 2015 (bottom half colored gray) and 2016 (top colored half white)

	N	S	P	K	Mg	Ca	Na	Fe	Al	Mn	B	Cu	Zn	Symptoms
N		0.0658***	0.0480***	-0.0415*	-0.0672***	-0.0859***	-0.0080	0.0652	0.0641	0.0120	-0.0461	0.0517	-0.0397	-0.1198*
S	**0.4852*****		0.0199***	0.0305***	0.0017	0.0765*	-0.0731	0.0321***	0.0371***	0.1283***	0.0417*	-0.0193***	-0.0467*	0.0418
P	**0.3674*****	**0.4856*****		-0.0114***	0.0550	-0.1348*	0.0052	0.0552***	0.0576***	-0.1298	0.0374*	-0.0359***	0.0253***	0.0853***
K	0.0941	**0.3154**	**0.4310*****		-0.0942	-0.2209***	-0.1474	0.0666***	0.0534***	0.0605	0.0696	-0.1061***	-0.0009***	**0.5834*****
Mg	**-0.3552*****	-0.0979***	0.0773	-0.1182		-0.1738***	0.2105**	0.2126	0.2129*	-0.1709***	0.1796***	0.1364***	0.0309***	-0.0991***
Ca	**-0.3393*****	0.0370	-0.1476*	**-0.4599*****	**0.4414*****		0.0375	-0.1189**	-0.1209*	-0.0240***	-0.0218	-0.0793	-0.0304*	0.1717***
Na	-0.0182	0.0276	0.1362*	0.0430	0.3175***	0.1414*		0.1264**	0.1150**	-0.1383	0.1282	-0.0229**	-0.0619***	0.1822***

Fe	-0.1512*	0.0970	0.0127	0.1419*	0.1663*	0.0729	0.1847*		0.9675***	0.2478**	-0.0181*	0.0619***	0.1048*	0.2284***
Al	-0.2220**	0.0546	0.0497	0.1566*	0.2220**	0.0702	0.1601*	0.9675***		0.0254**	0.0148*	0.0237***	-0.0184*	0.0453***
Mn	-0.2626***	0.1324*	-0.1350*	-0.0504	**0.3666***	**0.5618***	0.1167	0.2478***	0.2520***		0.0805*	-0.0297*	0.0509**	0.0648*
B	0.0947	0.1494*	0.0769	-0.0654	0.1767*	0.0432	0.1391*	-0.0181	-0.0173	0.0484		-0.0117	-0.1072	0.0312
Cu	-0.0277	0.0096	0.0276	0.0768	0.0945	-0.0068	0.1787*	0.0619	0.0642	0.0416	0.0361		-0.0546***	-0.1629***
Zn	-0.0547	0.1616*	0.2889***	0.2367**	0.0981	0.1142	0.1744*	0.1048	0.1217	0.1591*	-0.0184	0.1399*		0.7816
Symptoms	-0.2275**	-0.2259	0.0370	0.0583	**0.6146***	0.0059	0.2973***	0.2149**	0.2406***	0.0881	0.0519	0.0270	-0.0874	

Significant at ≤0.0001 (***) 0.001 (**) and 0.05 (*).

Supplemental Table 3: Pearson correlation coefficients among nutrients in 2015 (year 1) and 2016 (year 2).

	N	S	P	K	MG	CA	NA	F	AL	MN	B	CU	ZN
N2	0.2661***	0.1060	0.2055**	0.1459*	-0.1263*	-0.2169**	0.0564	-0.0251	-0.0546	-0.1464	-0.0365	0.0065	0.1053
S2	0.0658	0.3093***	0.0751	0.1188	0.0241	-0.0308	-0.0370	0.0998	0.0755	0.0893	0.0144	0.0854	0.0455
P2	0.0480**	0.0199	0.2303***	0.1285*	0.1955*	-0.1749*	0.0848	0.0944	0.1172	-0.0938	0.0342	0.0409	-0.0164
K2	-0.0415	0.0305	-0.0114	0.4060***	0.1103	-0.2242**	0.0331	0.2083**	0.2030*	0.1249*	-0.0090	0.0848	-0.0632
MG2	-0.0672	0.0017	0.0550	-0.0942	0.4557***	0.0803	0.0422	-0.0432	-0.0310	0.0917	0.0067	0.0208	0.0383
CA2	-0.0859	0.0765	-0.1348*	-0.2209**	-0.1738*	0.3187***	-0.2094**	-0.2086**	-0.2325**	0.1092	-0.1478*	-0.0232	0.0816
NA2	-0.0080	-0.0731	0.0052	-0.1474*	0.2105**	0.0375	0.4026***	0.0713	0.0415	0.0109	0.1002	-0.0302	-0.0177
FE2	0.0652	0.0321	0.0552	0.0666	0.2126	-0.1189	0.1264	0.2721***	0.2872***	0.1290*	0.1099	0.0644	-0.1364*
AL2	0.0641	0.0371	0.0576	0.0534	0.2129**	-0.1209	0.1150	0.2687***	0.2908***	0.1154	0.1229	0.0524	-0.1470*
MN2	0.0120	0.1283*	-0.1298*	0.0605	-0.1709*	-0.0240	-0.1383*	0.0512	0.0254	0.3479***	-0.1317*	0.0688	0.0582
B2	-0.0461	0.0417	0.0374	0.0696	0.1796*	-0.0218	0.1282*	-0.0195	0.0148	0.0805	0.4417***	0.0616	0.0023
CU2	0.0517	-0.0193	-0.0359	-0.1061	0.1364*	-0.0793	-0.0229	0.0436	0.0237	-0.0297	-0.0117	-0.0199	-0.1006
ZN2	-0.0397	-0.0467	0.0253	-0.0009	0.0309	-0.0304	-0.0619	-0.0202	-0.0184	0.0509	-0.1072	-0.0546	0.2086**

Significant at ≤0.0001 (***) 0.001 (**) and 0.05 (*).

Supplemental Table 4: Nutrient ratios associated with symptomatic (Symp) or non symptomatic (No Symp) plants in 2015 and 2016

	2015		2016	
	**Symp**	**No Symp**	**Symp**	**No Symp**
Mg/Ca	0.37*	0.26	0.24*	0.34
Al/Fe	0.59*	0.6	0.66	0.65
Al/Mg	377.27*	304.618	457.11*	375.13
Mg/K	0.99*	0.73	1.37*	1.18
Mg/N	0.43*	0.28	0.64*	0.51
N/Na	134.40*	91.2	46.60*	34.16
Na/K	0.03*	0.02	0.08	0.07
Na/Mg	0.03	0.03	0.07*	0.05

* indicates significant at P = 0.05

Supplemental Table 5: SNPs associated with Mg, Na, marginal chlorosis (MC), stunting (SL), and symptoms in 2015 (year 1) and 2016 (year 2) using Thompson Seedless gene annotation.

Ion	SNP	Chromosome	Position	Major Allele Frequency	SNP effect	FDR Adjusted P-values	Year
MC	S1_17012584	1	17012584	0.32489451	0.22659066	0.01667885	1
	S3_149721	3	149721	0.21308017	0.33281749	0.00056555	1
	S3_265316	3	265316	0.32700422	0.26375046	0.02374226	1
	S3_921272	3	921272	0.21729958	0.25154293	0.0046545	1
	S3_951268	3	951268	0.33966245	0.21896912	0.02403079	1
	S3_951388	3	951388	0.33122363	0.22961638	0.02847315	1
	S3_1940787	3	1940787	0.33333333	0.23369988	0.02403079	1
	S3_2800042	3	2800042	0.21729958	0.24541523	0.0055526	1
	S3_3366649	3	3366649	0.23628692	0.26617543	0.00243863	1
	S3_3366650	3	3366650	0.25316456	0.29170471	0.00056555	1
	S3_3708527	3	3708527	0.20886076	0.25551943	0.01667885	1
	S3_3762073	3	3762073	0.35232068	0.23271696	0.03941491	1
	S3_4305461	3	4305461	0.33755274	0.25830352	0.02283101	1
	S3_5273832	3	5273832	0.28691983	0.40386373	0.00019026	1
	S3_5336687	3	5336687	0.2257384	0.24659421	0.01667885	1
	S3_5569343	3	5569343	0.33544304	0.32242211	0.0025915	1
	S3_7859939	3	7859939	0.34388186	0.22103727	0.04314381	1
	S3_8105992	3	8105992	0.32700422	0.24961136	0.03941491	1
	S3_8106968	3	8106968	0.32911392	0.25310763	0.03941491	1
	S3_8107007	3	8107007	0.32489451	0.25282443	0.03941491	1
	S3_8415247	3	8415247	0.21518987	0.20905837	0.01012007	1
	S3_11599143	3	11599143	0.33966245	0.31090086	0.0025915	1
	S3_12359712	3	12359712	0.21097046	0.24237338	0.02403079	1
	S3_13316810	3	13316810	0.32911392	0.25657243	0.02746531	1
	S3_13577682	3	13577682	0.33544304	0.30991258	0.0055526	1
	S3_13577823	3	13577823	0.10759494	-0.2410146	0.0226152	1
	S3_13598294	3	13598294	0.33544304	0.21280628	0.02403079	1
	S3_14277010	3	14277010	0.33966245	0.22315704	0.03941491	1
	S3_14277018	3	14277018	0.33966245	0.22315704	0.03941491	1
	S3_14422878	3	14422878	0.21308017	0.26616849	0.01022214	1
	S3_14422903	3	14422903	0.33122363	0.25380778	0.02361311	1
	S3_14537967	3	14537967	0.26582278	0.29221882	0.00181432	1

	S3_15151065	3	15151065	0.20886076	0.26874619	0.00060935	1
	S3_17285477	3	17285477	0.24472574	-0.1779894	0.02418664	1
	S3_18408625	3	18408625	0.33544304	0.29786181	0.00675532	1
	S3_18664986	3	18664986	0.33544304	0.29837124	0.001007	1
	S3_18665025	3	18665025	0.33544304	0.29837124	0.001007	1
	S3_18838966	3	18838966	0.21097046	0.23317317	0.02844832	1
	S3_21716906	3	21716906	0.33122363	0.29674515	0.01084248	1
	S3_21825918	3	21825918	0.22995781	0.30575095	0.00041602	1
	S3_21825925	3	21825925	0.22995781	0.30575095	0.00041602	1
	S3_21825966	3	21825966	0.25738397	0.30791427	0.00039485	1
	S3_22457867	3	22457867	0.20675105	0.23251597	0.01220527	1
	S3_23241926	3	23241926	0.33966245	0.26967825	0.01136632	1
	S3_23242021	3	23242021	0.33544304	0.22987758	0.03084853	1
	S3_23242056	3	23242056	0.33544304	0.22987758	0.03084853	1
	S3_23257153	3	23257153	0.35021097	0.25169307	0.02371533	1
	S10_9101907	10	9101907	0.32278481	0.24333347	0.01245173	1
	S10_9101926	10	9101926	0.32278481	0.24333347	0.01245173	1
	S10_21319026	10	21319026	0.2721519	0.24909282	0.02403079	1
	S10_21319060	10	21319060	0.2721519	0.24909282	0.02403079	1
	S11_16654495	11	16654495	0.33122363	0.23112125	0.00235	1
	S12_17501478	12	17501478	0.30801688	0.22422583	0.02403079	1
	S14_22076746	14	22076746	0.26160338	0.17652429	0.02847315	1
	S18_19991790	18	19991790	0.29535865	0.24715794	0.01942903	1
	S18_19991805	18	19991805	0.29535865	0.24715794	0.01942903	1
	S18_29587371	18	29587371	0.28902954	-0.2016059	0.0067376	1
	S1_17928457	1	17928457	0.21097046	0.24985258	0.01447473	2
	S1_17928525	1	17928525	0.20675105	0.22616589	0.02473869	2
	S2_13405930	2	13405930	0.30801688	0.1964977	0.02794864	2
	S3_149721	3	149721	0.21308017	0.28332339	0.00319514	2
	S3_265316	3	265316	0.32700422	0.22134437	0.04671235	2
	S3_951268	3	951268	0.33966245	0.20430519	0.01928891	2
	S3_951388	3	951388	0.33122363	0.23582758	0.01293689	2
	S3_1940825	3	1940825	0.21940928	0.22291945	0.01609706	2
	S3_2800042	3	2800042	0.21729958	0.21037971	0.01447473	2
	S3_3366649	3	3366649	0.23628692	0.23660555	0.00558214	2
	S3_3366650	3	3366650	0.25316456	0.24592358	0.00319514	2
	S3_3708527	3	3708527	0.20886076	0.22094818	0.02794864	2
	S3_3755967	3	3755967	0.20886076	0.20468927	0.04746561	2
	S3_4805266	3	4805266	0.33333333	0.19529754	0.04746561	2
	S3_5273832	3	5273832	0.28691983	0.29069364	0.0060768	2

	S3_5336687	3	5336687	0.2257384	0.28237956	0.00319514	2
	S3_5569343	3	5569343	0.33544304	0.25697467	0.01447473	2
	S3_8415247	3	8415247	0.21518987	0.1714754	0.02918022	2
	S3_10836535	3	10836535	0.22995781	0.16520756	0.04452054	2
	S3_11143564	3	11143564	0.19620253	0.23030251	0.02794864	2
	S3_11599143	3	11599143	0.33966245	0.25913183	0.01293689	2
	S3_12359712	3	12359712	0.21097046	0.21291395	0.03528829	2
	S3_13577682	3	13577682	0.33544304	0.24854465	0.0194313	2
	S3_14422878	3	14422878	0.21308017	0.23478359	0.01447473	2
	S3_14422903	3	14422903	0.33122363	0.27046072	0.00453437	2
	S3_14422928	3	14422928	0.32489451	0.21364317	0.01810773	2
	S3_14537967	3	14537967	0.26582278	0.23418652	0.01447473	2
	S3_15151065	3	15151065	0.20886076	0.20459273	0.01447473	2
	S3_18408625	3	18408625	0.33544304	0.23876115	0.02473869	2
	S3_18664986	3	18664986	0.33544304	0.22846081	0.01447473	2
	S3_18665025	3	18665025	0.33544304	0.22846081	0.01447473	2
	S3_21716906	3	21716906	0.33122363	0.24464525	0.02473869	2
	S3_21762832	3	21762832	0.23628692	0.19296171	0.02473869	2
	S3_21825918	3	21825918	0.22995781	0.25053381	0.00319514	2
	S3_21825925	3	21825925	0.22995781	0.25053381	0.00319514	2
	S3_21825966	3	21825966	0.25738397	0.25288375	0.00319514	2
	S3_23241926	3	23241926	0.33966245	0.22078443	0.02794864	2
	S3_23257153	3	23257153	0.35021097	0.20963799	0.04746561	2
	S10_9101907	10	9101907	0.32278481	0.22247125	0.01447473	2
	S10_9101926	10	9101926	0.32278481	0.22247125	0.01447473	2
	S10_21319026	10	21319026	0.2721519	0.22299517	0.02918022	2
	S10_21319060	10	21319060	0.2721519	0.22299517	0.02918022	2
	S16_8791589	16	8791589	0.26582278	0.18539035	0.01614494	2
	S18_29587371	18	29587371	0.28902954	-0.1717003	0.01822124	2
	S18_32070700	18	32070700	0.24683544	0.18323085	0.0194313	2
	S18_32070713	18	32070713	0.25316456	0.19130682	0.01447473	2
	S19_15553095	19	15553095	0.22362869	0.1971494	0.0194313	2
	S1_17928457	1	17928457	0.21097046	0.24985258	0.01447473	2
	S1_17928525	1	17928525	0.20675105	0.22616589	0.02473869	2
	S2_13405930	2	13405930	0.30801688	0.1964977	0.02794864	2
	S3_149721	3	149721	0.21308017	0.28332339	0.00319514	2
	S3_265316	3	265316	0.32700422	0.22134437	0.04671235	2
	S3_951268	3	951268	0.33966245	0.20430519	0.01928891	2
	S3_951388	3	951388	0.33122363	0.23582758	0.01293689	2
	S3_1940825	3	1940825	0.21940928	0.22291945	0.01609706	2

	S3_2800042	3	2800042	0.21729958	0.21037971	0.01447473	2
	S3_3366649	3	3366649	0.23628692	0.23660555	0.00558214	2
	S3_3366650	3	3366650	0.25316456	0.24592358	0.00319514	2
	S3_3708527	3	3708527	0.20886076	0.22094818	0.02794864	2
	S3_3755967	3	3755967	0.20886076	0.20468927	0.04746561	2
	S3_4805266	3	4805266	0.33333333	0.19529754	0.04746561	2
	S3_5273832	3	5273832	0.28691983	0.29069364	0.0060768	2
	S3_5336687	3	5336687	0.2257384	0.28237956	0.00319514	2
	S3_5569343	3	5569343	0.33544304	0.25697467	0.01447473	2
	S3_8415247	3	8415247	0.21518987	0.1714754	0.02918022	2
	S3_10836535	3	10836535	0.22995781	0.16520756	0.04452054	2
	S3_11143564	3	11143564	0.19620253	0.23030251	0.02794864	2
	S3_11599143	3	11599143	0.33966245	0.25913183	0.01293689	2
	S3_12359712	3	12359712	0.21097046	0.21291395	0.03528829	2
	S3_13577682	3	13577682	0.33544304	0.24854465	0.0194313	2
	S3_14422878	3	14422878	0.21308017	0.23478359	0.01447473	2
	S3_14422903	3	14422903	0.33122363	0.27046072	0.00453437	2
	S3_14422928	3	14422928	0.32489451	0.21364317	0.01810773	2
	S3_14537967	3	14537967	0.26582278	0.23418652	0.01447473	2
	S3_15151065	3	15151065	0.20886076	0.20459273	0.01447473	2
	S3_18408625	3	18408625	0.33544304	0.23876115	0.02473869	2
	S3_18664986	3	18664986	0.33544304	0.22846081	0.01447473	2
	S3_18665025	3	18665025	0.33544304	0.22846081	0.01447473	2
	S3_21716906	3	21716906	0.33122363	0.24464525	0.02473869	2
	S3_21762832	3	21762832	0.23628692	0.19296171	0.02473869	2
	S3_21825918	3	21825918	0.22995781	0.25053381	0.00319514	2
	S3_21825925	3	21825925	0.22995781	0.25053381	0.00319514	2
	S3_21825966	3	21825966	0.25738397	0.25288375	0.00319514	2
	S3_23241926	3	23241926	0.33966245	0.22078443	0.02794864	2
	S3_23257153	3	23257153	0.35021097	0.20963799	0.04746561	2
	S10_9101907	10	9101907	0.32278481	0.22247125	0.01447473	2
	S10_9101926	10	9101926	0.32278481	0.22247125	0.01447473	2
	S10_21319026	10	21319026	0.2721519	0.22299517	0.02918022	2
	S10_21319060	10	21319060	0.2721519	0.22299517	0.02918022	2
	S16_8791589	16	8791589	0.26582278	0.18539035	0.01614494	2
	S18_29587371	18	29587371	0.28902954	-0.1717003	0.01822124	2
	S18_32070700	18	32070700	0.24683544	0.18323085	0.0194313	2
	S18_32070713	18	32070713	0.25316456	0.19130682	0.01447473	2
	S19_15553095	19	15553095	0.22362869	0.1971494	0.0194313	2
Mg	S3_21825918	3	21825918	0.22995781	0.06990251	0.00678768	1

	S3_21825925	3	21825925	0.22995781	0.06990251	0.00678768	1
	S3_21825966	3	21825966	0.25738397	0.06540355	0.01138848	1
	S3_3366650	3	3366650	0.25316456	0.06020626	0.04001324	1
Na	S11_16152632	11	16152632	0.30168776	-0.0858618	0.04865443	1
	S13_13525320	13	13525320	0.33966245	-0.1241383	0.04865443	1
	S13_13525321	13	13525321	0.33966245	-0.1241383	0.04865443	1
	S13_13525326	13	13525326	0.33966245	-0.1241383	0.04865443	1
	S18_25745134	18	25745134	0.33122363	0.13522212	0.04865443	1
	S18_25745143	18	25745143	0.33122363	0.13522212	0.04865443	1
	S11_1383030	11	1383030	0.34599156	0.11948124	0.01150161	2
	S11_11635675	11	11635675	0.35021097	0.12785527	0.01150161	2
	S11_16152632	11	16152632	0.30168776	-0.0864402	0.01150161	2
	S11_19913960	11	19913960	0.34599156	-0.0874211	0.0107111	2
	S12_14804000	12	14804000	0.21097046	-0.1089728	0.01725993	2
	S18_25745134	18	25745134	0.33122363	0.12717595	0.01298471	2
	S18_25745143	18	25745143	0.33122363	0.12717595	0.01298471	2
SL	S3_149721	3	149721	0.21308017	0.33946165	0.00321538	1
	S3_265316	3	265316	0.32700422	0.28700503	0.02325618	1
	S3_921272	3	921272	0.21729958	0.26027	0.01181936	1
	S3_2032882	3	2032882	0.20886076	0.2281167	0.03281731	1
	S3_2032883	3	2032883	0.21097046	0.2304321	0.03838502	1
	S3_2032939	3	2032939	0.20253165	0.23181374	0.03281731	1
	S3_2800042	3	2800042	0.21729958	0.23180025	0.03281731	1
	S3_3184421	3	3184421	0.33333333	0.18195281	0.0447322	1
	S3_3366650	3	3366650	0.25316456	0.22682294	0.04048876	1
	S3_3708527	3	3708527	0.20886076	0.2949255	0.01152956	1
	S3_4784875	3	4784875	0.20675105	0.22838344	0.03281731	1
	S3_5273832	3	5273832	0.28691983	0.36704589	0.00321538	1
	S3_5569343	3	5569343	0.33544304	0.30514975	0.01795723	1
	S3_5755118	3	5755118	0.35232068	0.18595655	0.0447322	1
	S3_10174220	3	10174220	0.20675105	0.25660224	0.03281731	1
	S3_11599143	3	11599143	0.33966245	0.30218	0.01337565	1
	S3_12359712	3	12359712	0.21097046	0.27459279	0.01969509	1
	S3_13316810	3	13316810	0.32911392	0.29200147	0.01969509	1
	S3_13577682	3	13577682	0.33544304	0.29973869	0.02117013	1
	S3_14422878	3	14422878	0.21308017	0.2829439	0.01485584	1
	S3_14537967	3	14537967	0.26582278	0.25758042	0.03117463	1
	S3_14638969	3	14638969	0.20464135	0.23848655	0.03281731	1
	S3_15151065	3	15151065	0.20886076	0.24392656	0.01255123	1
	S3_18408625	3	18408625	0.33544304	0.29347872	0.02117013	1

	S3_18664986	3	18664986	0.33544304	0.24077264	0.04425129	1
	S3_18665025	3	18665025	0.33544304	0.24077264	0.04425129	1
	S3_18838966	3	18838966	0.21097046	0.24791605	0.03838502	1
	S3_21716906	3	21716906	0.33122363	0.32520194	0.01152956	1
	S3_21825918	3	21825918	0.22995781	0.28060315	0.00766035	1
	S3_21825925	3	21825925	0.22995781	0.28060315	0.00766035	1
	S3_21825966	3	21825966	0.25738397	0.28325842	0.00600405	1
	S3_22457867	3	22457867	0.20675105	0.22388941	0.04048876	1
	S3_23241926	3	23241926	0.33966245	0.2618259	0.03281731	1
	S3_23242021	3	23242021	0.33544304	0.25010719	0.03281731	1
	S3_23242056	3	23242056	0.33544304	0.25010719	0.03281731	1
	S3_23257153	3	23257153	0.35021097	0.25580925	0.03923276	1
	S18_29587371	18	29587371	0.28902954	-0.2485896	0.00321538	1
	S1_17928457	1	17928457	0.21097046	0.24871813	0.02812041	2
	S1_17928525	1	17928525	0.20675105	0.22914848	0.04045198	2
	S2_13405930	2	13405930	0.30801688	0.20421323	0.03311981	2
	S3_149721	3	149721	0.21308017	0.32309111	0.00090024	2
	S3_265316	3	265316	0.32700422	0.25904701	0.02812041	2
	S3_921272	3	921272	0.21729958	0.22576551	0.01196371	2
	S3_951268	3	951268	0.33966245	0.21150045	0.02812041	2
	S3_951388	3	951388	0.33122363	0.21596282	0.03985745	2
	S3_1066739	3	1066739	0.21518987	0.19176613	0.02812041	2
	S3_1066750	3	1066750	0.20886076	0.19417887	0.02812041	2
	S3_1940787	3	1940787	0.33333333	0.22666266	0.02931871	2
	S3_1940825	3	1940825	0.21940928	0.21754486	0.03311981	2
	S3_2019299	3	2019299	0.19409283	0.22131001	0.04059856	2
	S3_2032882	3	2032882	0.20886076	0.19446222	0.04455843	2
	S3_2032939	3	2032939	0.20253165	0.19890423	0.04059856	2
	S3_2800042	3	2800042	0.21729958	0.23040632	0.00928362	2
	S3_3366649	3	3366649	0.23628692	0.25272535	0.00423453	2
	S3_3366650	3	3366650	0.25316456	0.27414261	0.00121981	2
	S3_3708527	3	3708527	0.20886076	0.28292448	0.00423453	2
	S3_4305461	3	4305461	0.33755274	0.24877468	0.02812041	2
	S3_4791541	3	4791541	0.20464135	0.20719061	0.03690097	2
	S3_4926121	3	4926121	0.28691983	0.17510822	0.03550842	2
	S3_4926123	3	4926123	0.28691983	0.17510822	0.03550842	2
	S3_5273832	3	5273832	0.28691983	0.38140452	0.00018347	2
	S3_5336687	3	5336687	0.2257384	0.29031631	0.00147413	2
	S3_5569343	3	5569343	0.33544304	0.31700306	0.00423453	2
	S3_5755118	3	5755118	0.35232068	0.16681274	0.03938057	2

	S3_7859939	3	7859939	0.34388186	0.21706591	0.04059856	2
	S3_8105992	3	8105992	0.32700422	0.24657795	0.0382301	2
	S3_8106968	3	8106968	0.32911392	0.24793745	0.03938057	2
	S3_8107007	3	8107007	0.32489451	0.24728348	0.03985745	2
	S3_8415247	3	8415247	0.21518987	0.21532144	0.00472126	2
	S3_10174220	3	10174220	0.20675105	0.22145334	0.03938057	2
	S3_10836535	3	10836535	0.22995781	0.18150863	0.02812041	2
	S3_11599143	3	11599143	0.33966245	0.31573881	0.00234523	2
	S3_12359712	3	12359712	0.21097046	0.26620894	0.00772624	2
	S3_13316810	3	13316810	0.32911392	0.25203148	0.02997139	2
	S3_13577682	3	13577682	0.33544304	0.30458901	0.00772299	2
	S3_13598294	3	13598294	0.33544304	0.20937041	0.02812041	2
	S3_14277010	3	14277010	0.33966245	0.21966695	0.03762031	2
	S3_14277018	3	14277018	0.33966245	0.21966695	0.03762031	2
	S3_14422878	3	14422878	0.21308017	0.26033418	0.00978336	2
	S3_14422903	3	14422903	0.33122363	0.2469158	0.02812041	2
	S3_14537967	3	14537967	0.26582278	0.27142637	0.00423453	2
	S3_14638969	3	14638969	0.20464135	0.20816951	0.03550368	2
	S3_15151065	3	15151065	0.20886076	0.25665943	0.00117259	2
	S3_18408625	3	18408625	0.33544304	0.29409045	0.00772624	2
	S3_18664986	3	18664986	0.33544304	0.24281331	0.01196371	2
	S3_18665025	3	18665025	0.33544304	0.24281331	0.01196371	2
	S3_18838966	3	18838966	0.21097046	0.23011363	0.02812041	2
	S3_21716906	3	21716906	0.33122363	0.29474037	0.00978336	2
	S3_21762832	3	21762832	0.23628692	0.20001132	0.03261204	2
	S3_21825918	3	21825918	0.22995781	0.30804543	0.00018347	2
	S3_21825925	3	21825925	0.22995781	0.30804543	0.00018347	2
	S3_21825966	3	21825966	0.25738397	0.30679038	0.00018347	2
	S3_22457867	3	22457867	0.20675105	0.23005432	0.00978336	2
	S3_23241926	3	23241926	0.33966245	0.26288037	0.01196371	2
	S3_23257153	3	23257153	0.35021097	0.2410554	0.02997139	2
	S10_9101907	10	9101907	0.32278481	0.22060387	0.02812041	2
	S10_9101926	10	9101926	0.32278481	0.22060387	0.02812041	2
	S10_21319026	10	21319026	0.2721519	0.26432832	0.00978336	2
	S10_21319060	10	21319060	0.2721519	0.26432832	0.00978336	2
	S10_28026244	10	28026244	0.33544304	0.19642459	0.0244633	2
	S11_16654495	11	16654495	0.33122363	0.17569753	0.03570541	2
	S12_17501478	12	17501478	0.30801688	0.22002896	0.0244633	2
	S16_8791589	16	8791589	0.26582278	0.18416558	0.02878896	2
	S18_19991790	18	19991790	0.29535865	0.26311194	0.00772299	2

	S18_19991805	18	19991805	0.29535865	0.26311194	0.00772299	2
	S18_29587371	18	29587371	0.28902954	-0.1907808	0.00883184	2
	S18_32070700	18	32070700	0.24683544	0.1767133	0.04059856	2
	S18_32070713	18	32070713	0.25316456	0.18260382	0.03261204	2
Symp	S3_149721	3	149721	0.21308017	0.36419565	0.00149684	1
	S3_265316	3	265316	0.32700422	0.30184279	0.01730875	1
	S3_921272	3	921272	0.21729958	0.27182084	0.00837379	1
	S3_2032882	3	2032882	0.20886076	0.23990008	0.02443169	1
	S3_2032939	3	2032939	0.20253165	0.24436033	0.02423876	1
	S3_2800042	3	2800042	0.21729958	0.24640477	0.01730875	1
	S3_3366650	3	3366650	0.25316456	0.2561027	0.0149644	1
	S3_3708527	3	3708527	0.20886076	0.30823981	0.00837379	1
	S3_4784875	3	4784875	0.20675106	0.23123744	0.03253264	1
	S3_5273832	3	5273832	0.28691983	0.40843832	0.00149684	1
	S3_5569343	3	5569343	0.33544304	0.3260476	0.01079826	1
	S3_10174220	3	10174220	0.20675106	0.26783533	0.02443169	1
	S3_11599143	3	11599143	0.33966245	0.32413799	0.00837379	1
	S3_12359712	3	12359712	0.21097046	0.2890058	0.01200528	1
	S3_13316810	3	13316810	0.32911392	0.30502883	0.0149644	1
	S3_13577682	3	13577682	0.33544304	0.31692615	0.01528289	1
	S3_14422878	3	14422878	0.21308017	0.3000511	0.00837379	1
	S3_14537967	3	14537967	0.26582279	0.29158571	0.00837379	1
	S3_14638969	3	14638969	0.20464135	0.25152138	0.02131426	1
	S3_15151065	3	15151065	0.20886076	0.29015535	0.00149684	1
	S3_18408625	3	18408625	0.33544304	0.31060458	0.0149644	1
	S3_18664986	3	18664986	0.33544304	0.25486735	0.03238005	1
	S3_18665025	3	18665025	0.33544304	0.25486735	0.03238005	1
	S3_18838966	3	18838966	0.21097046	0.28444197	0.01149192	1
	S3_21716906	3	21716906	0.33122363	0.34071368	0.00837379	1
	S3_21825918	3	21825918	0.22995781	0.31010264	0.00186685	1
	S3_21825925	3	21825925	0.22995781	0.31010264	0.00186685	1
	S3_21825966	3	21825966	0.25738397	0.312007	0.00149684	1
	S3_22457867	3	22457867	0.20675106	0.26453392	0.00890808	1
	S3_23241926	3	23241926	0.33966245	0.28227404	0.02164565	1
	S3_23242021	3	23242021	0.33544304	0.29320071	0.00837379	1
	S3_23242056	3	23242056	0.33544304	0.29320071	0.00837379	1
	S3_23257153	3	23257153	0.35021097	0.2673552	0.04043637	1
	S11_16654495	11	16654495	0.33122363	0.22964664	0.01019004	1
	S18_29587371	18	29587371	0.28902954	-0.2316787	0.00593366	1
	S1_17928457	1	17928457	0.210970464	0.248718128	0.028120409	2

	S1_17928525	1	17928525	0.206751055	0.229148482	0.040451983	2
	S2_13405930	2	13405930	0.308016878	0.204213226	0.033119807	2
	S3_21825966	3	21825966	0.257383966	0.306790375	0.00018347	2
	S3_5273832	3	5273832	0.286919831	0.381404519	0.00018347	2
	S3_21825918	3	21825918	0.229957806	0.308045432	0.00018347	2
	S3_21825925	3	21825925	0.229957806	0.308045432	0.00018347	2
	S3_149721	3	149721	0.213080169	0.32309111	0.00090024	2
	S3_15151065	3	15151065	0.208860759	0.256659427	0.001172592	2
	S3_3366650	3	3366650	0.253164557	0.274142613	0.001219805	2
	S3_5336687	3	5336687	0.225738397	0.290316307	0.001474127	2
	S3_11599143	3	11599143	0.339662447	0.315738812	0.002345231	2
	S3_5569343	3	5569343	0.335443038	0.317003058	0.004234527	2
	S3_3708527	3	3708527	0.208860759	0.282924477	0.004234527	2
	S3_3366649	3	3366649	0.23628692	0.252725345	0.004234527	2
	S3_14537967	3	14537967	0.265822785	0.27142637	0.004234527	2
	S3_8415247	3	8415247	0.215189873	0.215321441	0.004721257	2
	S3_13577682	3	13577682	0.335443038	0.304589007	0.007722992	2
	S3_12359712	3	12359712	0.210970464	0.266208945	0.007726242	2
	S3_18408625	3	18408625	0.335443038	0.294090452	0.007726242	2
	S3_2800042	3	2800042	0.217299578	0.230406322	0.009283624	2
	S3_14422878	3	14422878	0.213080169	0.260334179	0.009783359	2
	S3_22457867	3	22457867	0.206751055	0.230054315	0.009783359	2
	S3_21716906	3	21716906	0.331223629	0.294740368	0.009783359	2
	S3_921272	3	921272	0.217299578	0.225765512	0.011963712	2
	S3_23241926	3	23241926	0.339662447	0.262880368	0.011963712	2
	S3_18664986	3	18664986	0.335443038	0.242813308	0.011963712	2
	S3_18665025	3	18665025	0.335443038	0.242813308	0.011963712	2
	S3_14422903	3	14422903	0.331223629	0.246915796	0.028120409	2
	S3_13598294	3	13598294	0.335443038	0.209370411	0.028120409	2
	S3_265316	3	265316	0.327004219	0.259047012	0.028120409	2
	S3_1066750	3	1066750	0.208860759	0.194178869	0.028120409	2
	S3_10836535	3	10836535	0.229957806	0.181508634	0.028120409	2
	S3_18838966	3	18838966	0.210970464	0.230113626	0.028120409	2
	S3_4305461	3	4305461	0.337552743	0.248774684	0.028120409	2
	S3_951268	3	951268	0.339662447	0.211500453	0.028120409	2
	S3_1066739	3	1066739	0.215189873	0.191766132	0.028120409	2
	S3_1940787	3	1940787	0.333333333	0.226662665	0.029318709	2
	S3_13316810	3	13316810	0.329113924	0.252031476	0.02997139	2
	S3_23257153	3	23257153	0.35021097	0.241055397	0.02997139	2
	S3_21762832	3	21762832	0.23628692	0.200011323	0.032612039	2

	S3_1940825	3	1940825	0.219409283	0.217544856	0.033119807	2
	S3_14638969	3	14638969	0.20464135	0.20816951	0.03550368	2
	S3_4926121	3	4926121	0.286919831	0.175108216	0.035508421	2
	S3_4926123	3	4926123	0.286919831	0.175108216	0.035508421	2
	S3_4791541	3	4791541	0.20464135	0.20719061	0.036900973	2
	S3_14277010	3	14277010	0.339662447	0.219666949	0.037620314	2
	S3_14277018	3	14277018	0.339662447	0.219666949	0.037620314	2
	S3_8105992	3	8105992	0.327004219	0.246577952	0.038230098	2
	S3_8106968	3	8106968	0.329113924	0.247937453	0.039380573	2
	S3_5755118	3	5755118	0.352320675	0.166812736	0.039380573	2
	S3_10174220	3	10174220	0.206751055	0.221453342	0.039380573	2
	S3_8107007	3	8107007	0.324894515	0.247283484	0.039857452	2
	S3_951388	3	951388	0.331223629	0.215962817	0.039857452	2
	S3_7859939	3	7859939	0.343881857	0.217065913	0.040598557	2
	S3_2019299	3	2019299	0.194092827	0.221310012	0.040598557	2
	S3_2032939	3	2032939	0.202531646	0.198904235	0.040598557	2
	S3_2032882	3	2032882	0.208860759	0.194462216	0.044558432	2
	S10_21319026	10	21319026	0.272151899	0.264328317	0.009783359	2
	S10_21319060	10	21319060	0.272151899	0.264328317	0.009783359	2
	S10_28026244	10	28026244	0.335443038	0.196424588	0.0244633	2
	S10_9101907	10	9101907	0.32278481	0.220603874	0.028120409	2
	S10_9101926	10	9101926	0.32278481	0.220603874	0.028120409	2
	S11_16654495	11	16654495	0.331223629	0.17569753	0.035705409	2
	S12_17501478	12	17501478	0.308016878	0.220028962	0.0244633	2
	S16_8791589	16	8791589	0.265822785	0.18416558	0.028788959	2
	S18_19991790	18	19991790	0.29535865	0.263111942	0.007722992	2
	S18_19991805	18	19991805	0.29535865	0.263111942	0.007722992	2
	S18_29587371	18	29587371	0.289029536	-0.190780811	0.008831843	2
	S18_32070713	18	32070713	0.253164557	0.18260382	0.032612039	2
	S18_32070700	18	32070700	0.246835443	0.176713304	0.040598557	2

Supplemental Table 6: SNPs located in genic regions and associated with Mg, Na, marginal chlorosis (MC), stunting (SL), and symptoms (Symp) in 2015 and 2016 using the Thompson Seedless annotation.

SNP	GENE	TRAIT	YEAR
S10_19692199	g1087	Symp	2015, 2016
S3_149721	g1405	MC	2015, 2016
		SL	2015, 2016
		Symp	2015, 2016
S3_265316	g1407	MC	2015, 2016
		SL	2015, 2016
		Symp	2015, 2016
S3_921272	g1462	MC	2015
		SL	2015, 2016
		Symp	2015, 2016
S3_2019299	g1518	SL	2016
		Symp	2016
S3_2032939	g1523	SL	2015, 2016
		Symp	2015, 2016
S3_2032882	g1523	Symp	2015, 2016
		SL	2015, 2016
S3_2032883	g1523	SL	2015
S3_2800042	g1599	MC	2015, 2016
		SL	2015, 2016
		Symp	2015, 2016
S3_3184421	g1621	SL	2015
S3_3366649	g1632	MC	2015, 2016
		SL	2016
		Mg	2015
S3_3366650	g1632	MC	2015
		Mg	2015
		SL	2015, 2016
		Symp	2015, 2016
S3_3755967	g1649	MC	2016
		Symp	2016
S3_3762073	g1649	MC	2015
S3_4305461	g1676	MC	2015
		SL	2016
		Symp	2016
S3_4773182	g1689	Symp	2016
S3_4784875	g1690	SL	2015

		Symp	2015
S3_4791541	g1691	SL	2016
		Symp	2016
S3_4805266	g1692	MC	2016
S3_5336687	g1724	MC	2015, 2016
		SL	2016
		Symp	2016
S3_5569343	g1736	MC	2015, 2016
		SL	2015, 2016
		Symp	2015, 2016
S3_5755118	g1741	SL	2015, 2016
		Symp	2016
S3_7859939	g1859	MC	2015
		SL	2016
		Symp	2016
S3_8104173	g1868	Symp	2016
S3_8105992	g1868	MC	2015
		SL	2016
		Symp	2016
S3_8106968	g1868	MC	2015
		SL	2016
		Symp	2016
S3_8107007	g1868	MC	2015
		SL	2016
		Symp	2016
S3_8105992	g1868	MC	2015
S3_8415247	g1882	MC	2015, 2016
		SL	2016
		Symp	2016
S3_10174220	g1999	SL	2015, 2016
		Symp	2015, 2016
S3_10836535	g2020	MC	2016
		SL	2016
		Symp	2016
S3_11143564	g2026	MC	2016
S3_11599143	g2046	MC	2015, 2016
		SL	2015, 2016
		Symp	2015, 2016
S3_12359712	g2086	MC	2015, 2016
		SL	2015, 2016

		Symp	2015, 2016
		MC	2015, 2016
S3_13577682	g2137	MC	2015, 2016
		SL	2015, 2016
		Symp	2015, 2016
S3_13577823	g2137	MC	2015
S3_14277010	g2174	MC	2015
		SL	2016
		Symp	2016
S3_14277018	g2174	MC	2015
		SL	2016
		Symp	2016
S3_14537967	g2185	MC	2015, 2016
		SL	2015, 2016
		Symp	2015, 2016
S3_15151065	g2217	MC	2015, 2016
		SL	2015, 2016
		Symp	2015, 2016
S3_18838966	g2363	MC	2015
		SL	2015, 2016
		Symp	2015, 2016
S3_22457867	g2581	MC	2015
		SL	2015, 2016
		Symp	2015, 2016
S3_23241926	g2622	MC	2015, 2016
		SL	2015, 2016
		Symp	2015, 2016
S3_23242021	g2622	MC	2015
		SL	2015
		Symp	2015
S3_23242056	g2622	MC	2015
		Symp	2015
		SL	2015
S1_17012584	g26984	MC	2015
		SL	2015, 2016
S10_9101907	g575	MC	2015, 2016
		Symp	2016
		SL	2016
S10_9101926	g575	MC	2015, 2016
		SL	2016

		Symp	2016
S11_11635675	g13071	Na	2016

Supplemental Table 7: SNPs located in genic regions and associated with Mg, Na, marginal chlorosis (MC), stunting (SL), and symptoms (Symp) in 2015 and 2016 using the PN40024 v3 annotation.

SNP	Gene	Trait	Year
S3_2846002	Vitvi03g00243	SL	2015, 2016
		Symp	2016
S3_2876678	Vitvi03g00247	SL	2015, 2016
S3_4196400	Vitvi03g00380	MC	2016
		SL	2015, 2016
S3_4208958	Vitvi03g00384	MC	2016
		SL	2015, 2016
		Symp	2015, 2016
S3_4209015	Vitvi03g00384	MC	2016
		SL	2015, 2016
		Symp	2015, 2016
S3_4637832	Vitvi03g00430	MC	2015, 2016
		SL	2015, 2016
		Symp	2015, 2016
S3_5653914	Vitvi03g00520	MC	2015, 2016
		SL	2015, 2016
		Symp	2015, 2016
S3_5852953	Vitvi03g00534	MC	2016
		SL	2015, 2016
		Symp	2015, 2016
S3_5885203	Vitvi03g00537	MC	2016
S3_5961430	Vitvi03g00540	MC	2016
S3_5986778	Vitvi03g00543	MC	2015, 2016
		SL	2015, 2016
		Symp	2015, 2016
S3_6167883	Vitvi03g00560	MC	2015, 2016
		SL	2015, 2016
		Symp	2015, 2016
S3_6554413	Vitvi03g00583	MC	2015, 2016
		SL	2015, 2016
		Symp	2015, 2016
S3_6823070	Vitvi03g00603	MC	2015, 2016
		Mg	2015
		SL	2015, 2016
		Symp	2015, 2016
S3_6823120	Vitvi03g00603	MC	2016
S3_7009508	Vitvi03g00617	MC	2016
S3_7815436	Vitvi03g00688	Symp	2015,2016
S3_7815488	Vitvi03g00688	Symp	2015,2016
S3_7097770	Vitvi03g00626	MC	2016
S3_8516174	Vitvi03g00724	MC	2016
		SL	2015, 2016
		Symp	2016
S3_9107785	Vitvi03g00756	MC	2015, 2016
		SL	2015, 2016
S3_9286732	Vitvi03g00772	MC	2015, 2016
		Mg	2015
		SL	2015, 2016
S3_9374358	Vitvi03g00777	MC	2015, 2016
		SL	2015, 2016
		Symp	2015, 2016

S3_9441309	Vitvi03g00783	MC	2015, 2016
		SL	2015, 2016
S3_11054913	Vitvi03g00846	MC	2016
S3_11308538	Vitvi03g00855	MC	2015, 2016
		SL	2015, 2016
		Symp	2015, 2016
S3_11368101	Vitvi03g00858	MC	2015, 2016
		SL	2015, 2016
S3_12779089	Vitvi03g00925	MC	2015, 2016
		SL	2015, 2016
S3_14475933	Vitvi03g00997	MC	2016
		SL	2015, 2016
S3_14476028	Vitvi03g00997	MC	2015
		SL	2015
		Symp	2015
S3_14786293	Vitvi03g01012	MC	2015, 2016
		SL	2015, 2016
		Symp	2015, 2016
S3_16246430	Vitvi03g01067	MC	2015, 2016
		SL	2015, 2016
S3_16701286	Vitvi03g01088	MC	2016
		MC	2016
		SL	2016
		Symp	2016
S3_16707340	Vitvi03g01088	MC	2016
S3_16774264	Vitvi03g01092	MC	2016
		SL	2016
		Symp	2016
S3_16774395	Vitvi03g01092	MC	2015, 2016
		SL	2015, 2016
		Symp	2016
S3_18739154	Vitvi03g01193	SL	2015
		Symp	2015
S3_16247276	Vitvi03g01318	MC	2016
		SL	2016
		Symp	2016
S3_4201002	Vitvi03g01518	MC	2015, 2016
		SL	2015, 2016
S3_6106914	Vitvi03g01581	MC	2015, 2016
		SL	2015, 2016
S3_7076477	Vitvi03g01606	MC	2015, 2016
		Mg	2015
		SL	2015, 2016
S3_8895549	Vitvi03g01649	MC	2015, 2016
		Mg	2015
		SL	2015, 2016
S3_9889752	Vitvi03g01681	MC	2016
S3_10848055	Vitvi03g01704	SL	2015
S3_12277516	Vitvi03g01727	MC	2016
S3_15171032	Vitvi03g01769	MC	2015, 2016
		SL	2015, 2016
S3_15171570	Vitvi03g01769	MC	2015, 2016
		SL	2015, 2016
S3_16473090	Vitvi03g01792	MC	2016
		SL	2015, 2016
		Symp	2015, 2016
S3_17225576	Vitvi03g01812	MC	2016
		SL	2016
		Symp	2016

S3_18840198	Vitvi03g01841	SL	2015
S6_20771993	Vitvi06g01435	MC	2016
		SL	2016
		Symp	2016
S11_7516783	Vitvi11g00662	SL	2016
		Symp	2016
S11_12185252	Vitvi11g00916	Na	2016
S11_16113932	Vitvi11g01100	Na	2016
S11_19320005	Vitvi11g01270	Na	2016
S11_19319929	Vitvi11g01270	Na	2016
S11_18313262	Vitvi11g01649	Na	2016
S11_19307737	Vitvi11g01681	Na	2016
S11_19311299	Vitvi11g01682	Na	2016
S17_12265971	Vitvi17g01006	Na	2016
